# Inflammatory cytokines and a diverse cervicovaginal microbiota associate with cervical dysplasia in a cohort of Hispanics living in Puerto Rico

**DOI:** 10.1371/journal.pone.0284673

**Published:** 2023-12-08

**Authors:** Eduardo Tosado-Rodríguez, Loyda B. Mendez, Ana M. Espino, Stephanie Dorta-Estremera, Edna E. Aquino, Josefina Romaguera, Filipa Godoy-Vitorino

**Affiliations:** 1 Department of Microbiology and Medical Zoology, School of Medicine, Medical Sciences Campus, University of Puerto Rico, Carolina, Puerto Rico, United States of America; 2 University Ana G. Méndez, Carolina Campus, Carolina, Puerto Rico, United States of America; 3 Cancer Biology, Comprehensive Cancer Center University of Puerto Rico, Carolina, Puerto Rico, United States of America; 4 Department of OBGYN, School of Medicine, Medical Sciences Campus, University of Puerto Rico, Carolina, Puerto Rico, United States of America; Universidad San Francisco de Quito, ECUADOR

## Abstract

Cervical cancer (CC) is women’s fourth most common cancer worldwide. A worrying increase in CC rates in Hispanics suggests that besides Human papillomavirus infections, there may be other cofactors included in the epithelial microenvironment that could play a role in promoting the disease. We hypothesized that the cervical microbiome and the epithelial microenvironment favoring inflammation is conducive to disease progression in a group of Hispanics attending gynecology clinics in Puerto Rico. Few studies have focused on the joint microbiota and cytokine profile response in Hispanics outside the US, especially regarding the development of precancerous lesions. We aimed to investigate the relationship between the cervicovaginal microbiome and inflammation in Hispanic women living in PR while considering cervical dysplasia and HPV genotype risk. Cervical samples collected from 91 participants coming to gynecology clinics in San Juan, underwent 16S rRNA genes (V4 region) profiling, and cytokines were measured using Luminex MAGPIX technology. Cytokines were grouped as inflammatory (IL-1β, TNFα, IFNγ, IL-6), anti-inflammatory (IL- 4, IL-10, TGFβ1), and traffic-associated (IL-8, MIP1a, MCP1, IP10). They were related to microbes via an inflammation scoring index based on the quartile and tercile distribution of the cytokine’s concentration. We found significant differences in the diversity and composition of the microbiota according to HPV type according to carcinogenic risk, cervical disease, and cytokine abundance. Community State Types (CSTs) represents a profile of microbial communities observed within the vaginal microbiome ecological niche, and *Lactobacillus-*depleted CST IV had ~ 90% dominance in participants with high-grade squamous intraepithelial lesions and high-risk HPV. The increasing concentration of pro-inflammatory cytokines was associated with a decrease in *L*. *crispatus*. In contrast, dysbiosis-associated bacteria such as *Gardnerella*, *Prevotella*, *Atopobium* concomitantly increased with pro-inflammatory cytokines. Our study highlights that the cervical microbiota of Hispanics living in Puerto Rico is composed mostly of diverse CST profiles with decreased *Lactobacillus* and is associated with a higher pro-inflammatory environment. The joint host-microbe interaction analyses via cytokine and microbiota profiling have very good translational potential.

## Introduction

Cervical cancer (CC) is the 4th most common cancer among women and the 4th leading cause of cancer death in females worldwide [1]. Although CC rates have decreased in the USA [2], the incidence and mortality from cervical cancer mainly occur in low-resource settings (~ >85%) [3]. According to the Pan American Health Organization for 2012, 80% of American deaths due to CC occur in Latin America and the Caribbean [4]. Puerto Rico (PR) has the highest age-adjusted incidence of CC in the US, with notably higher incidence from 2001 to 2017, increasing from 9.2 to 13 per 100,000 person-years [5]. PR also has a 79.3% CC screening rate that does not achieve CDC’s screening recommendations of 93% [6] posing a strain on health systems in the island [7].

Persistent infection with oncogenic Human Papillomavirus (HPV) is the most important cause of CC. HPV infections range from 90–100% prevalence in cervical abnormalities, from high-grade squamous intraepithelial lesion (HGSIL) to cancer [8], where the viral genome integration often modulates the host cell cycle promoting neoplasia progression [9,10]. However, other factors influence cervical disease progression, including lifestyle, genetic predisposition, immunosuppression, early onset of sexual activities, and many pregnancies and deliveries [11–13]. Nevertheless, why only a fraction of women acquires a long-lasting, persistent high-risk HPV (HR-HPV) that puts them at risk for developing Cervical Intraepithelial Neoplasia (CIN) remains uncertain. One potential explanation for this variation could be the cervicovaginal microbiota and its related processes. Recent studies suggest an association between certain bacterial community types of the vaginal microbiota, HPV infection, and cervical disease [14–16]. Additionally, processes like bacterial vaginosis, cervical inflammation, and increased vaginal pH are all known to affect persistence and progression of cervical lesions [17].

Women of different ethnic groups differ in their vaginal microbiome signatures [18]. The cervicovaginal microbiome has been characterized into five different community state types (CST) according to the dominance of *Lactobacillus* species. This concept was introduced in 2011 by Ravel et al. to categorize vaginal microbial communities among reproductive-age women [18]. CST-I refers to communities dominated by *L*. *crispatus* (CST-I), CST-II by *L*. *gasseri*, CST-III by *L*. *iners*, CST-V by *L*. *jensenii* (CST-V), or non-*Lactobacillus* dominated and highly diverse profiles such as CST-IV. Furthermore, CST-IV is often characterized by three sub-CSTs: 1) CST IV-A has a high relative abundance of Candidatus *Lachnocurva vaginae* and a moderate relative abundance of *G*. *vaginalis* and *Atopobium vaginae*, 2) CST IV-B also has a moderate relative abundance of *G*. *vaginalis* and *Atopobium vaginae*, while 3) CST IV-C has low relative abundance of *Lactobacillus* spp., as well as low levels of *G*. *vaginalis*, *A*. *vaginae*, and *Ca*. *L*. *vaginae* with high facultative and strictly anaerobic bacteria [19]. While *Lactobacillus* dominates the cervicovaginal microbiome of Caucasians, Hispanic and black women have higher diversity and reduced *Lactobacillus* populations [18]. A previous study demonstrated that Hispanics living in PR have a different microbial profile than Caucasians, and that patients with high-risk HPV and CIN3 cervical lesions had dominant *Atopobium vaginae* and *Gardnerella vaginalis* [16,20]. However, these results could have been improved, as they identified microbiota using cervical swabs, failing to address the host response. Recent studies have highlighted the benefit of collecting cervical lavages to understand the extent of the dynamic host-microbe symbioses via proteomic, metabolomic, or cytokine profiling analyses [21–24].

We hypothesized that there would be changes in the cervical cytokine responses and the microbiota associated both with cervical disease and HPV carcinogenic risk types, and that markers of inflammation would be associated with a decrease in *Lactobacillus*. To test this hypothesis, we investigated the relationship between the cervicovaginal microbiome with cervical neoplasia and HPV infections and characterized the host immune profiles through profiling cytokine expression analyses from cervical lavages.

This study provides insight into specific bacterial taxonomic changes associated to pro-inflammatory states and cervical dysplasia and represents the first effort to combine microbiome and cytokine profiling in Hispanics living in Puerto Rico.

## Materials and methods

### Participant recruitment and sample collection

Females seeking gynecologic care at the University of Puerto Rico and San Juan City Hospital clinics were enrolled in our study. Only women who did not meet the exclusion criteria were recruited and evaluated. All women were HIV-negative. Exclusion criteria included: 1) Active urinary infections, 2) history of common urinary incontinence, 3) sexually transmitted diseases (STDs), 4) antibiotics taken in the prior month, 5) candidiasis, 6) being less than 21 and more than sixty years old, and 7) treatment for or suspicion of prior toxic shock syndrome. All subjects were informed of the study’s sampling procedure, risks, and benefits with written informed consent and a verbal explanation of the document. A detailed questionnaire was administered. Women recruitment and samples used in this study were collected between November 2017 and February 2020. DNA extractions and data analyses was done between March 2020 and October 2022.

This recruitment was conducted in compliance with the regulations of the Human Subjects Protection Office at the University of Puerto Rico, under the Institutional Review Board Medical Sciences Campus IRB protocol #1050114. This protocol was initially approved on June 24, 2014, and it has been continually renewed and remains in effect until 2024. The biosafety committee of the Medical Sciences Campus also approved the Protocol (IBC) # 94620. All procedures were done according to the Declaration of Helsinki.

A total of 91 women, 21–60 years old and able to provide informed consent, were enrolled in our study. Cervical samples were collected through speculum access during the gynecological examination by using cotton tip collection swabs. Sterile Catch-All Specimen Collection Swabs (Epicentre Biotechnologies, Madison WI, United States) were placed in the cervix, rotated along the lumen with a circular motion, and then placed in sterile 2 milliliters (mL) microtubes with cap. We collected approximately 10mL of cervical lavages (CVL) by injecting pure certified nuclease-free water (Growcells, Irvine, CA) into the vaginal canal. Collected lavages were stored in a clean 15mL collection tube and pH was measured with hydrion wide range pH paper strips short after sample collection at room temperature (Merck, Germany). All samples were kept from 1–4 hrs in a cooler with ice packs before transportation to the lab, where aliquots were prepared and further long-term storage was done at −80°C. Information from the medical records and questionnaires and the results from cervical cytology and pathology reports were gathered. These reports were used to categorize participants according to cervical disease status. Disease status was defined using cytology and HPV HR status (if NILM and HPV HR-was negative, no colposcopy was done). If patient cytology was abnormal (LGSIL or HGSIL) and or HPV HR-positive colposcopy was received and biopsy results were used in this case. In this study, the groupings included Negative for intraepithelial lesion or malignancy (NILM); low-grade squamous intraepithelial lesion (LGSIL), high-grade intraepithelial lesion (HGSIL).

### Cytokine detection and measurement with Bio-Plex immunoassay system

A total of 79 cervical lavages were tested in duplicate with a multiplex solid-based immunoassay (Bio-Plex Pro Human Cytokine 10-plex Assay, Bio-Rad) using the same volume of CVL across samples (80 uL) for the Luminex MAGPIX^®^ system. Cytokines/chemokines analyzed were IL-1β, IL-4, IL-6, IL-8, IL-10, TNF-α, IFNγ, IP10, MIP1, and MCP1. Also, 79 samples were tested in duplicate using Bio-Plex Pro TGF-β 3-plex immunoassay to quantify TGF-β 1 (BioRad, Hercules, CA, USA). We performed all analyses following the manufacturer’s recommendations.

### Cytokine/Chemokine profile analyses

Data were analyzed with the Bio-Plex Manager 6.1 software (BIO-RAD, Hercules, CA) using a 5-parameter logistic curve that was verified for normality before statistical testing [25]. Inflammation scores were assigned to each cytokine/chemokine for each sample using Fluorescent Intensity (FI) results. Scores were calculated by distributing samples across statistical quartiles or tertiles. If a sample was within the first quartile, a score of 1 was assigned, that is for all other three quartiles (score 1 < 0.25 < score 2 < 0.50 < score 3 < 0.75 < score 4 < 1). Cytokines were grouped according to their function on host physiology, including: pro-inflammatory (IL-1β, TNF-α, IFNγ, IL-6), anti-inflammatory (IL-4, IL-10, TGFβ1) and traffic-associated cytokines(IL-8, MIP1a, MCP1, IP10) [26,27]. With the scoring in quartiles, we also created a categorical variable, to which scores of 2 or 1 corresponded to low levels of cytokines and > 2 high levels. These ranked score assignments using the distribution of cytokines were directly related to the concentration (fluorescence intensity) of cytokines/chemokines in a sample. Therefore, we used them to combine microbial community analysis (16S rDNA profiles) with inflammation scores as a metadata variable (see methods section "Statistical Analyses of the Microbiota Profiles" for more details). This combination allowed us to detect specific taxa associated with altered cytokine profiles.

Cytokine profiles were evaluated according to cervical disease, CSTs, and HPV infection. These analyses were done using cytokine concentration (pg/mg protein) instead of the fluorescence intensity (FI) used for the inflammation scores. For comparisons between multiple groups, we used ANOVA, and for comparisons between two groups, we used the student’s *t*-test. All statistical analyses used GraphPad Prism 6.0 (San Diego, CA).

### DNA extraction and 16S rDNA sequencing

Genomic DNA extractions from swabs were performed with the Qiagen Power Soil Kit (QIAGEN LLC, Germantown Road, Maryland, USA) following an optimized protocol for these types of samples as described in a previous study [16]. DNA was quantified with a Qubit^®^ dsDNA HS (High Sensitivity) Assay (Waltham, Massachusetts, US) (ranging from 5-100ng/μL). An average of 10-30ng/μL genomic DNA was shipped to an outsourced sequencing facility.

DNA from cervical samples was normalized to 4nM during 16S library prep. The V4 hypervariable region of the 16S ribosomal RNA marker gene (~291bp) was amplified using universal bacterial primers: 515F (5’GTGCCAGCMGCCGCGGTAA3’) and 806R (5’GGACTACHVGGGTWTCTAAT3’) as in the Earth Microbiome Project (http://www.earthmicrobiome.org/emp-standard-protocols/16s/) [28]. The 16S-rRNA reads and corresponding sample metadata was deposited in QIITA study ID 12871 [29], and the raw sequences are available in the European Nucleotide Archive ENA Project under accession number EBI: ERP136546.

### HPV genotyping

We used a kit based on a highly sensitive short-polymerase chain reaction-fragment assay (Labo Biomedical Products, Rijswijk, The Netherlands, licensed Innogenetics technology). It uses SPF10 primers to amplify a 65-bp fragment of the L1 open reading frame of HPV genotypes, followed by a Reverse-Hybridization step to determine which HPV type is present by comparing to kit-provided controls. This assay allows for the identification of the following common mucosal HPV genotypes: 14 High-risk types including (HPV 16, 18, 31, 33, 35, 39,45, 51, 52, 56, 58, 59, 66 and 68/73) and 11 Low-risk types (6, 11, 34, 40,42, 43, 44, 53, 54,70, 74). Previous studies explain the method in detail [16,30].

In this paper we refer to HPV types as HPV type according to carcinogenic risk, hence we categorize it as HPV negative (HPV-), HPV+ Coinfection (having both low-risk and high-risk types) and HPV+ High risk exclusively.

### Quality control and sequence processing

Raw read pre-processing of demultiplexed files was done with a Phred offset of 33 and default parameters using split libraries FASTQ (QIIMEq2 1.9.1) in QIITA [29]. Sequences were trimmed to 250 bp, and for denoising we followed the deblurring workflow (deblur 1.1.0) [29,31]. For taxonomy assignment we used a modified Greengenes reference database (Greengenes_13.8) [32,33] with a minimum similarity threshold of 97%. The species table was downloaded for downstream analyses using a locally run version of QIIME [34]. Singletons, amplicon sequence variants (ASVs) with less than three reads, and sequences matching chloroplasts, mitochondria, and taxonomically unassigned sequences were removed from downstream analyses. All samples were rarefied to 7,400 reads per sample.

### Vaginal community state type nearest centroid classifier (VALENCIA)

The taxonomy of cervical microbial communities was sorted into categories termed community state types (CSTs). This categorization collapses a taxonomic profile into a single categorical variable, allowing data exploration and statistical modeling [19]. In addition, categorization allows unbiased analysis of small and sizable vaginal microbiota datasets, comparisons between datasets, and meta-analyses that combine multiple datasets. We used the data package at github.com/ravel-lab/VALENCIA to analyze the following standard parameters and categorize our cervicovaginal communities into different CST and sub-CST categories [19].

### Analyses of microbial community structure and diversity

For community-level analyses, we computed the pairwise Bray-Curtis distances (Beta diversity) which quantify the compositional dissimilarity between samples [35]. We visualized bacterial community composition and structure differences using non-metric multidimensional scaling (NMDS). Alpha diversity measure of Chao1 (richness), Shannon diversity index [36], and bar plots showing genus and species-level taxa were computed and plotted through R, using MicrobiomeAnalyst [37].

The most important metadata variables included in our study were cervical disease, HPV carcinogenic types and cytokine levels. Cervical disease was grouped as: a) negative for intraepithelial lesion or malignancy (NILM) who are HPV negative, b) NILM participants who are HPV positive were considered in the same category as participants with low-grade squamous intraepithelial lesion (LGSIL) and c) high-grade squamous intraepithelial lesion (HGSIL); HPV risk, was defined as HPV negative, HPV positive with co-infection (low and high-risk HPV simultaneously), and HPV positive high-risk (exclusively high-risk types). Microbiota was also associated with cytokine ranks (estimated through quartile distribution of the fluorescence scores as detected by Luminex MAGPIX^®^ system) and community state types (CSTs).

### Statistical analyses of the microbiota profiles

For beta diversity analyses, we tested if distances between sample groups were statistically significant using PERMANOVA, a non-parametric multivariate ANOVA statistical test [38]. Using a Bray-Curtis distance table, we used this test to compare ranked beta diversity distances between the different variables. These tests were done using the script "qiime diversity beta-group-significance” for each specific test in QIIME2 [34] with the distance matrix as the input file and 999 permutations [39]. Statistical analyses for alpha diversity were done using the script “qiime diversity alpha-group-significance” in QIIME2 [34], which uses a non-parametric t-test with Monte Carlo permutations. Cytokine fluorescence values (as logarithms) were associated to differentially abundant levels of *L*. *crispatus*, *L*. *iners*, *Sneathia*, *Gardnerella*, *Atopobium* and *Prevotella* using a linear mixed model (LMM) in R (ggplot and reshape2 libraries), correcting for age and body mass index (BMI). Differential abundance analysis was performed using amplicon sequence variants corresponding to *L*. *crispatus*, *L*. *iners*, *Sneathia*, *Atopobium*, *Gardnerella* and *Prevotella*. They were evaluated for their differential presence across the cytokine groups. Initially, a summary of each one of the ASVs of interest was used to perform groupings due to abundance. The groupings were separated as follows ASV 0–50 counts = very low abundance; ASV count 51–500 = low abundance; and >500 = high abundance. They were evaluated for their differential presence across the cytokine groups.

To note despite 91 women were recruited and samples collected the number of samples analyzed by the different methods differ. Specifically, for prevalence analysis of the metadata variables, we used the entire study cohort n = 91. In the case of cytokine profile analysis, we ended up with less samples due to cytokine concentration availability n = 79. When using fluorescence intensity in analyses where Transforming growth factors are included, we used n = 88. We chose fluorescence as a method to avoid altering the data before conducting correlation analyses. These same 88 samples were utilized for analyzing physiological status, cervical disease, and cytokines (pro-inflammatory, anti-inflammatory, and trafficking).

## Results

### Population description and community state types (CSTs) prevalence

A total of 91 women were included in the study. The mean age was 39 years old. Women had an average pH value of 5.49. Overall, 11% of our participants smoke, while 44% occasionally drink alcohol. The average age of first sexual encounter was 19 years old and the average number of sexual partners was 5. Most subjects were self-declared heterosexuals (98.0%). This study corresponds to an analyses of a subgroup of the nearly 300 patients which we recently characterized for only cervicovaginal microbiota and physiological status [40]. The distribution of the groups of samples according to cervical neoplasia severity included Negative for Intraepithelial Lesion or Malignancy (NILM) (N = 56) Low-Grade Squamous Intraepithelial Lesion (LGSIL) (N = 18), and High Grade Squamous Intraepithelial Lesion (HGSIL) (N = 17), with differential HPV status and cytokine ranks (Table 1 and S1 Table). HPV infections had a prevalence of 67.0% (including infections with any HPV type), with a higher prevalence of HPV+ (LR+HR) among women with LGSIL (94.4%, as these include NILM HPV+) and HSIL lesions (88.2%) (Table 1). Participants with HPV co-infections (LR+HR simultaneously) was primarily observed in participants with low-grade lesions (44.4%) (p = 0.040), and less abundant in the group with high-grade lesions (5.9%). There were no significant differences in the distribution of the BMI groups across cervical disease, with equal proportions of normal weight, overweight and obese women (Table 1). Across all women, cervical lesion prevalence was 38.5% (including LGSIL and HGSIL), Individually, LGSIL and HGSIL comprised 19.8% and 18.7% of the samples, respectively. CST1 mainly was present in 16% of the NILM cases and 22% LGSIL while not being present in HGSIL. In contrast, CST IV-B was only identified on participants with high-grade lesions (HGSIL) (p = 0.025) (Table 1). Prevalence analysis of CSTs per levels (high and low) of cytokine groups (pro-inflammatory, traffic-associated, and anti-inflammatory) revealed that CST IV-C is the more prevalent in all cytokines (S1 Fig, S2 Table).

**Table 1:** Characteristics of the selected cohort.

	NILM (N = 56)	LGSIL (N = 18)	HGSIL (N = 17)	TRUE (N = 91)	P-value *
**Age**					
Mean (SD)	40.4 (12.0)	39.6 (9.55)	33.9 (9.47)	39.1 (11.3)	0.213
Median [Min, Max]	38.5 [21.0, 60.0]	38.5 [25.0, 56.0]	33.0 [22.0, 56.0]	37.0 [21.0, 60.0]	
**BMI_Analysis**					
Normal	20 (35.7%)	5 (27.8%)	7 (41.2%)	32 (35.2%)	NS
Obese	18 (32.1%)	8 (44.4%)	5 (29.4%)	31 (34.1%)	
Overweight	18 (32.1%)	4 (22.2%)	5 (29.4%)	27 (29.7%)	
Not recorded	0 (0%)	1 (5.6%)	0 (0%)	1 (1.1%)	
**pH**					
Mean (SD)	5.50 (0.500)	5.36 (0.413)	5.60 (0.573)	5.49 (0.500)	0.486
Median [Min, Max]	5.50 [5.00, 7.00]	5.25 [5.00, 6.00]	5.50 [5.00, 6.50]	5.50 [5.00, 7.00]	
**HPV_Status**					
HPV-	27 (48.2%)	1 (5.6%)	2 (11.8%)	30 (33.0%)	
HPV+	29 (51.8%)	17 (94.4%)	15 (88.2%)	61 (67.0%)	2.48EE-4
**HPV_Risk**					
HPV-	**27 (48.2%)**	1 (5.6%)	2 (11.8%)	30 (33.0%)	0.003
Co-infection	7 (12.5%)	**8 (44.4%)**	1 (5.9%)	16 (17.6%)	0.040
High_Risk	22 (39.3%)	9 (50.0%)	**14 (82.4%)**	45 (49.5%)	0.016
**CST**					
I	9 (16.1%)	4 (22.2%)	0 (0%)	13 (14.3%)	
IV-A	12 (21.4%)	5 (27.8%)	5 (29.4%)	22 (24.2%)	
IV-C	35 (62.5%)	9 (50.0%)	8 (47.1%)	52 (57.1%)	
IV-B	0 (0%)	0 (0%)	**4 (23.5%)**	4 (4.4%)	0.025

*Kruskal-Wallis’s test for continuous and Fisher’s test for categorical variables.

### Cytokine changes according to high-grade lesions and community state types (CSTs)

**Fig 1:**
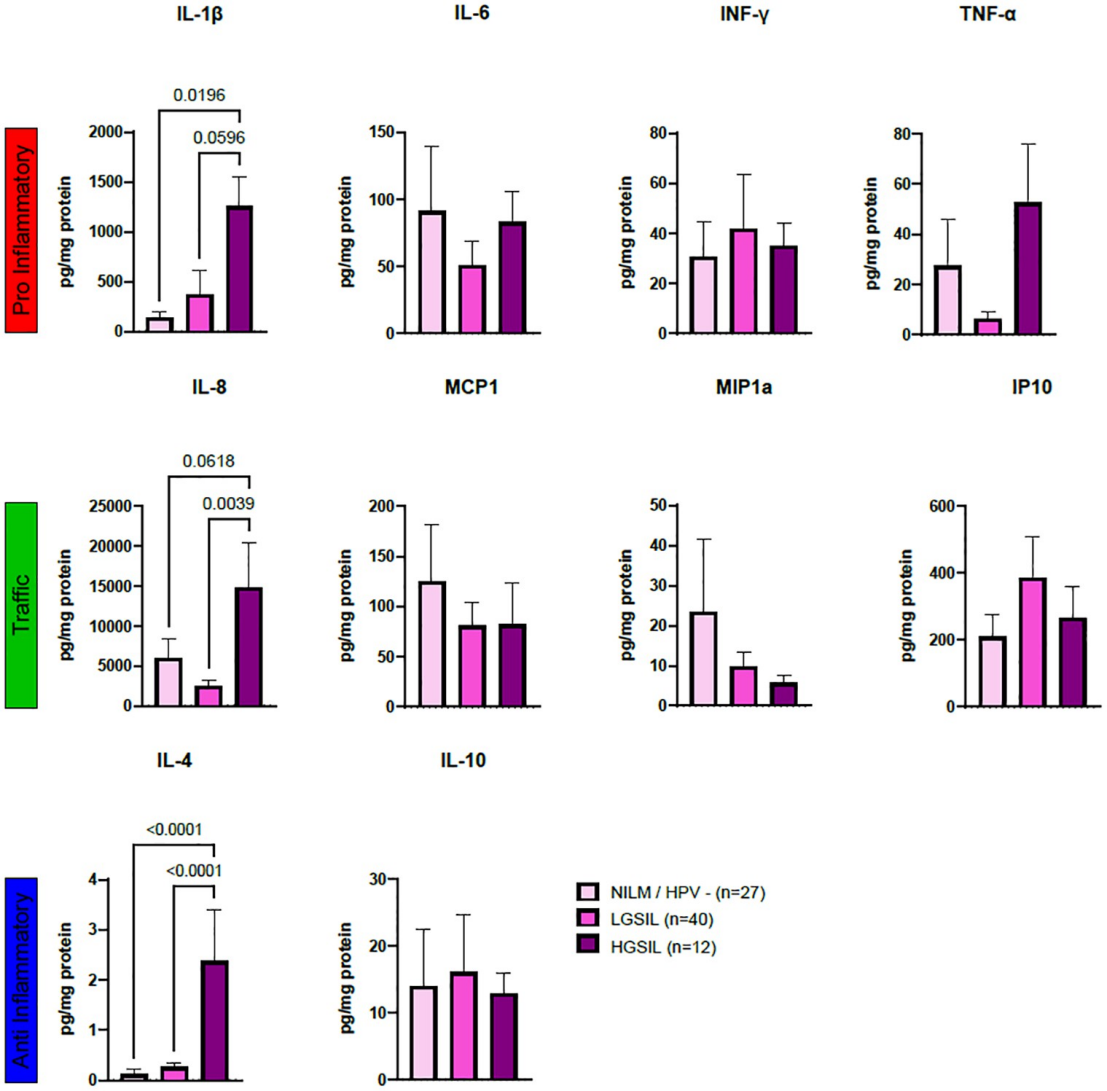
Cytokine profiles evaluated through LUMINEX and grouped according to their immunological role. Pro inflammatory (A) (IL-1β, TNFα, IFNγ, IL-6), anti-inflammatory **(B)** (IL-4, IL-10, TGFβ1) and traffic-associated cytokines **(C)** (IL-8, MIP1a, MCP1, IP10) evaluated through LUMINEX and plotted according to cervical disease. Cytokine concentrations (pg/mg protein) were used to compute multiple comparison analysis using ordinary one-way ANOVA with Tukey’s multiple comparisons test. Results were depicted in boxplots for cervical disease. Significant differences are highlighted by brackets and corresponding p-values.

Significant variations in cytokine concentrations (measured in pg/mg of protein) were observed in women with high-grade disease compared to healthy participants. Notably, there were significantly higher concentration levels in HGSIL compared to (NILM / HPV-; p = 0.019), and the values were nearly significant in participants with low-grade disease (p = 0.059) (see Fig 1). Additionally, IL-8, a cytokine associated with neutrophil recruitment, exhibited borderline significance in HGSIL when compared to NILM/HPV- (p = 0.061) and showed significant increases when compared to LGSIL (p = 0.003) (Fig 1). The anti-inflammatory cytokine IL-4 also displayed significant differences with an increase in HGSIL compared tod NILM/HPV- (p = 0.0618), as well as LGSIL (p = 0.0039). Although an analysis examining cytokines based on HPV risk was conducted, no significant differences were observed (refer to S2 Fig).

**Fig 2:**
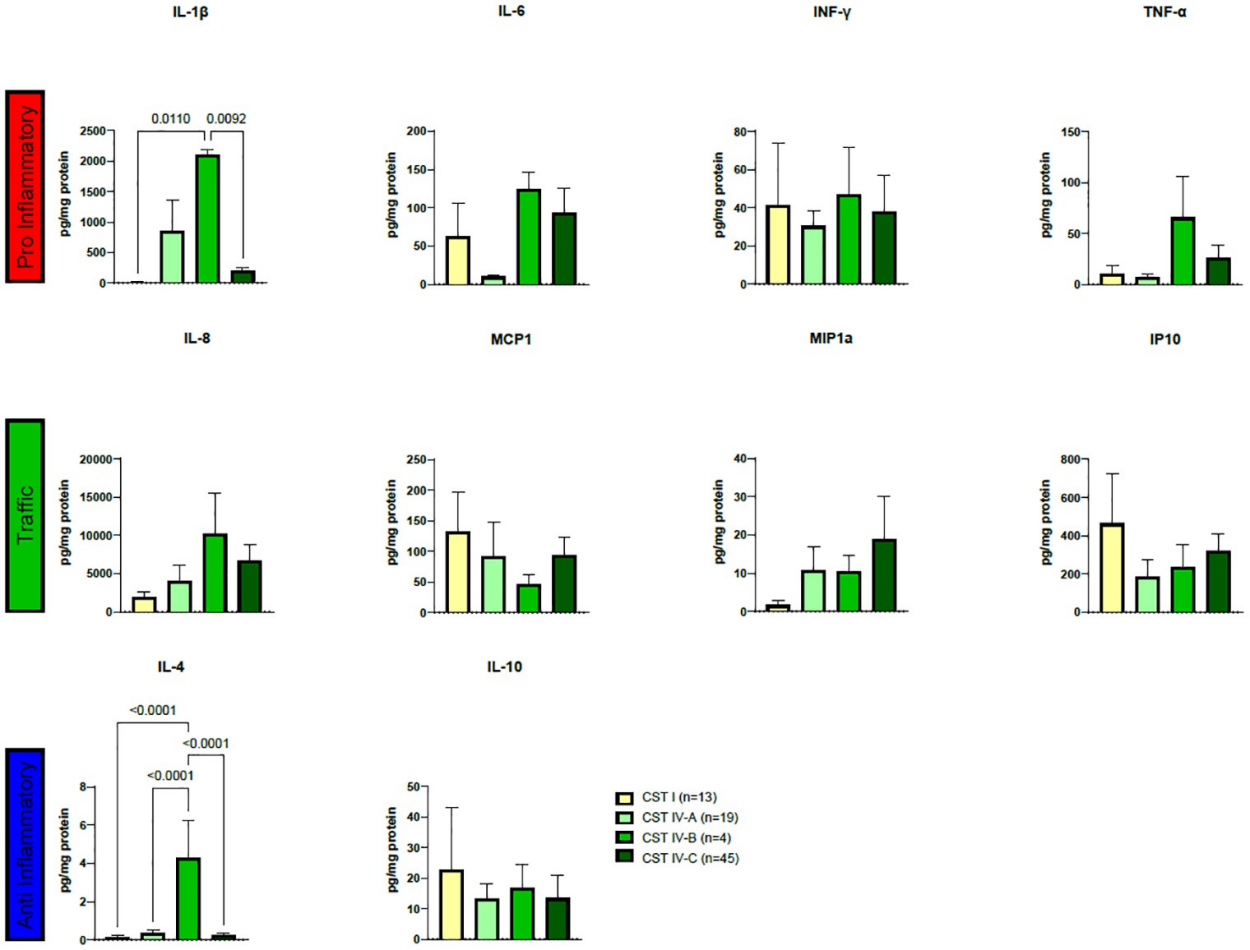
Cytokine profiles evaluated through LUMINEX, plotted according to CST classification. Cytokine concentrations (pg/mg protein) were used to compute multiple comparison analyses using ordinary one-way ANOVA with Tukey’s multiple comparisons tests. Results are depicted in boxplots for cervical disease, and brackets and corresponding p-values highlight significant differences.

When examining cytokines based on CSTs, notable distinctions in IL-1β and IL-4 concentrations were identified, see Fig 2. Regarding IL-1β, CST IV-B exhibited significantly elevated concentrations compared to CST I (p = 0.0110) and CST IV-C (p = 0.0092) (Fig 2). Similarly, IL-4 displayed significantly higher concentrations in CST IV-B than in CST I (p<0.0001), CST IV-A (p<0.0001), and CST IV-C (p<0.0001) (Fig 2). However, a distinct pattern of cytokine concentration was not observed within any of the groups (Fig 2).

### HPV infection and cervical disease are associated with altered bacterial community composition

A total of 2,067,074 high-quality reads of 16S rRNA sequences and 8,167 OTUs were subjected to analysis, with a rarefaction threshold set at 7,400 reads per sample. For most analyses the sample pool primarily consisted of women of reproductive age (n = 70) and menopausal women (n = 18). We examined the cytokine profiles related to menopausal status (S3A, S3B and S3C Fig). We observed no significant distinctions in cytokine profiles between menopausal and non-menopausal women. We analyzed beta and alpha diversity within these groups to explore whether alterations in cervicovaginal microbiota were associated with women’s physiological states. However, we found no significant differences in either beta diversity (Bray-Curtis) (S3D Fig) or alpha diversity (Shannon) (S3E Fig). The microbiota composition revealed no specific taxa linked to the assessed groups (S3F Fig). It is worth noting that all associations between cytokines and specific taxa were adjusted for age.

**Fig 3:**
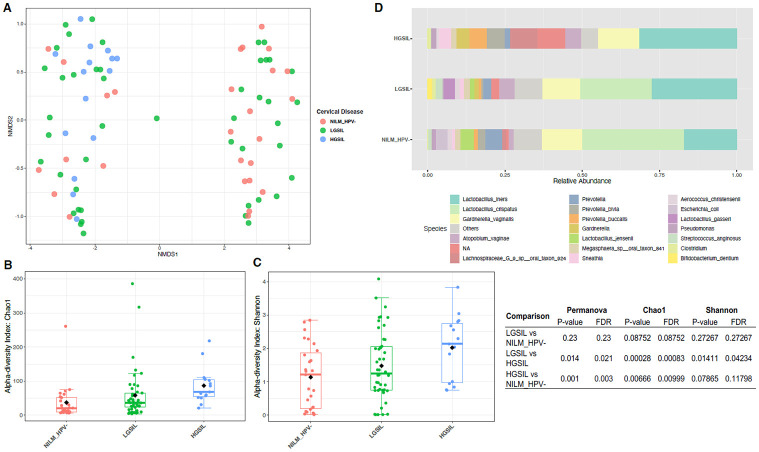
Diversity analyses according to cervical disease and HPV risk categories. Bray-Curtis analysis represented by Non-metric multidimensional scaling (NMDS) using cervical disease and HPV risk as metadata categories (A, D). Alpha diversity (Shannon) was calculated and depicted in Figs B and E. Bar Plots depicting relative abundance of bacteria at the species level for cervical disease (C) and HPV risk (F). Red asterisks mean significant differences among groups (p<0.05).

Furthermore, we assessed the differences in microbiota based on cervical disease, considering HPV infections. Beta diversity analyses related to cervical disease showed that the structure of the bacterial community significantly differed in cases of high-grade cervical disease (HGSIL) compared to healthy controls (NILM/HPV-) (p = 0.003) and when compared to low-grade cervical disease (LGSIL) (p = 0.021) (Fig 3A). Both richness (Chao1) and diversity (Shannon) concurrently increased with the severity of epithelial lesions. Chao1 demonstrated significant differences between NILM/HPV- and HGSIL (p = 0.009) and between LGSIL and HGSIL (p = 0.021) (Fig 3B). Shannon also revealed a significant increase in diversity in HGSIL compared to LGSIL (p = 0.042) (Fig 3C).

Regarding microbiota composition, NILM/HPV- and LGSIL participants exhibited similar profiles dominated by *Lactobacillus crispatus* and *Lactobacillus iners*. However, at the species level, we observed an increase in *L*. *iners* communities and a decrease in *L*. *crispatus* in LGSIL (Fig 3D). In contrast, participants with HGSIL showed alterations in *Lactobacillus* distribution, with disappearance of protective *L*. *crispatus* and an increase in *L*. *iners*, along with other taxa associated with dysbiosis such as Lachnospiraceae sp., *Prevotella bivia*, *Atopobium vaginae*, and *Gardnerella vaginalis* (Fig 3D).

### Association of bacterial taxa with cytokine levels

**Fig 4:**
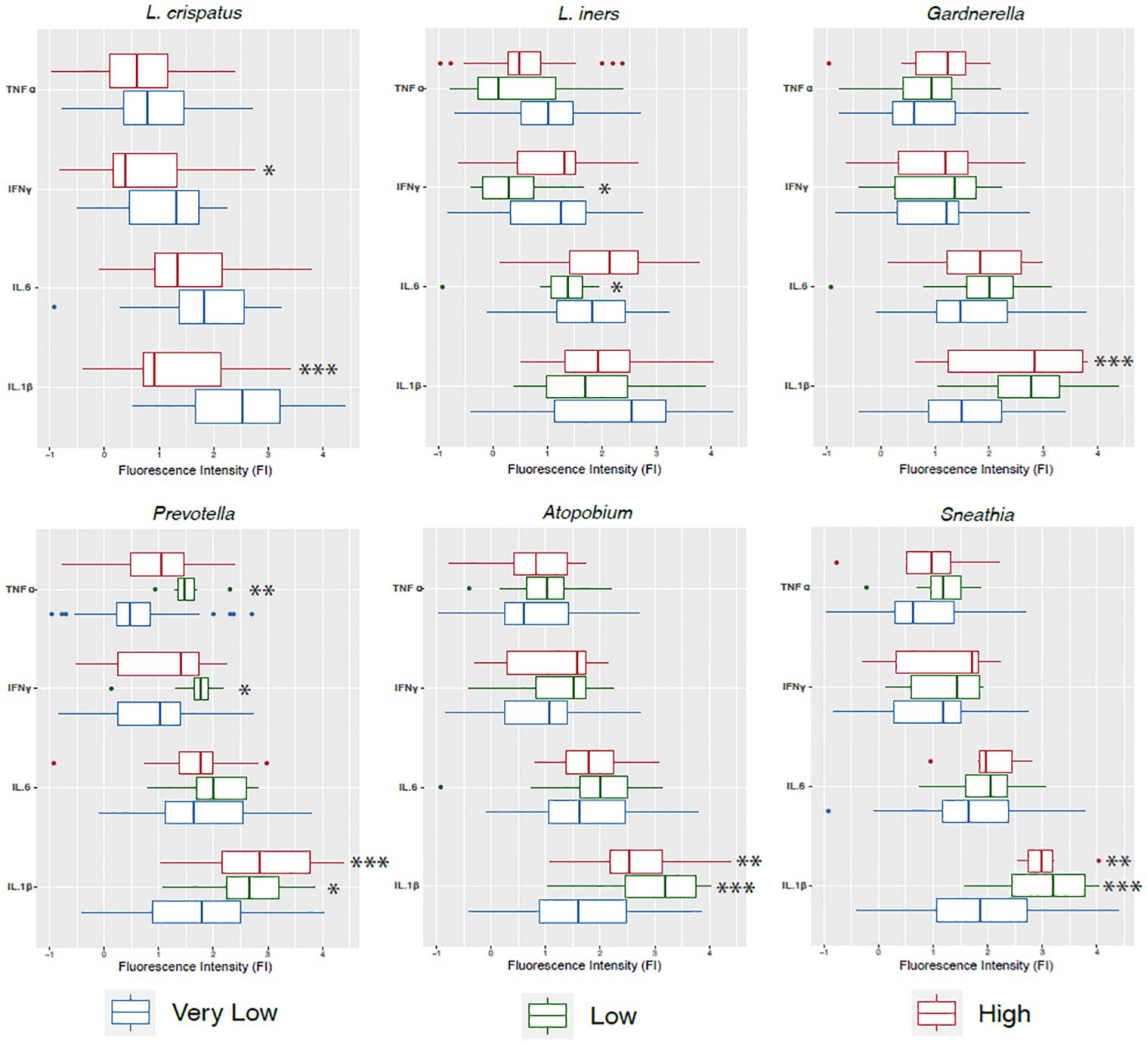
Association of key vaginal bacteria with levels of pro-inflammatory cytokines. Cytokine logarithm fluorescence values were associated to differentially abundant levels of *L*. *crispatus*, *L*. *iners*, *Sneathia*, *Gardnerella*, *Atopobium* and *Prevotella* using a linear mixed model (LMM), correcting for age and BMI. Differential abundance groupings were separated as follows ASV 0–50 counts = very low abundance; ASV count 51–500 = low abundance; and >500 = high abundance. * = 0.05; ** = 0.01; *** = 0.001.

In a balanced vaginal tract, *Lactobacillus* species impede the colonization of other bacteria that would otherwise cause infections. *L*. *Crispatus* produce lactic acid, bacteriocins and H_2_O_2_ acting as a chemical barrier [1]. Our linear mixed model revealed that high levels of *L*. *crispatus*, a key vaginal bacterium in eubiosis, were significantly associated to low concentrations of pro-inflammatory cytokines IL-1β (p = 4.18e-06) and IFNγ (p = 0.022) (Fig 4). Low levels of *L*. *iners* were significantly associated with lower levels of IL-6 (p = 0.021) and IFNγ (0.015), also pro-inflammatory cytokines (Fig 4). On the contrary, low levels of *L*. *iners* correlate with lower expression of IFNγ (p = 0.039) (Fig 4). High levels of anaerobic *Gardnerella*, were associated with higher fluorescence values of IL-1β (p = 1.62e-05) (Fig 4). Low abundance *of Prevotella* correlated with higher concentrations of IL-1β (p = 0.020), IFNγ (p = 0.011) and TNFα (p = 0.003) in our samples (Fig 4). Also, high concentrations of *Prevotella* correlated to IL-1β (p = 3.00e-05). Low (p = 8.34e-06) and high (p = 0.002) levels of *Atopobium*, a mostly pathogenic bacteria, were significantly associated with high levels of IL-1β. As for *Sneathia*, another dysbiosis associated taxa, for which low (p = 0.0009) and high (p = 0.006) abundances had a correlation with higher levels of IL-1β (Fig 4).

In community composition (beta diversity), X axis of the NMDS1 plot separated samples according to their cytokine levels (S4A–S4C Fig). We found changes in microbial community structure significantly associated with high levels of pro-inflammatory (p = 0.001), anti-inflammatory (p = 0.001) and traffic-associated cytokines (p = 0.019) (S4 Fig). When the levels of pro-inflammatory cytokines were low, alpha diversity (Shannon index) was significantly higher (p = 0.00008) (S4A Fig). High levels of anti-inflammatory cytokines associated to higher Shannon diversity (p = 0.000003) (S4B Fig). We found no significant differences in alpha diversity in traffic-associated cytokines (S4C Fig). Taxonomic abundance at species level showed that in the 3 cytokine groups, populations of *L*. *crispatus* decreases with higher cytokine levels. Participants with high levels of cytokines were dominated by *L*. *iners*, as *L*. *crispatus* nearly disappears with increased levels of cytokines. Additionally, this group of participants were dominated by *Prevotella sp*., *A*. *vaginae* and *G*. *vaginalis*. Simultaneously, *Lachnospiraceae sp*. was detected mainly on participants with High cytokine levels, confirming the dysbiosis typical of *Lactobacillus* depleted communities (S4A–S4C Fig).

## Discussion

Cervical cancer (CC) is a complex disease where the cervical environment is modified, including cytokine expression that leads to a local immunosuppression state [23]. More than 30 of the several human papillomavirus (HPV) subtypes cause genital tract infections. It is generally known that specific oncogenic (high-risk) HPV strains (~15 types) are linked to cervical cancer. Detecting HPV has limited effectiveness in predicting invasive cervical cancer since only a small number of women who test positive for HPV actually develop High-Grade Squamous Intraepithelial Lesions (HGSIL) [41,42]. Inflammation and the microbiome could be the missing link to understanding the cellular mechanisms leading to persistent HPV infection and dysplasia progression. Indeed, to provide an accurate diagnosis and prognosis of cervical disease progression, screening tests have limited sensitivity for cytology [43]. Our sensitive HPV genotyping method demonstrated that HPV prevalence in this cohort, recruited from gynecology clinics was 67.0% (encompassing infections with any HPV type) and that co-infections with any HPV serotype were primarily present in low-grade lesions (44.4%). Exclusively high-risk types were more prevalent in HGSIL (82.4%), a prevalence previously reported by our team [16] and Hispanic and Amerindian peoples [30]. This higher prevalence exceeds those reported for Costa Ricans [44], or women in Surinam [45], Nigeria [46], Turkey [47] or Italy [48]. This can be attributed to the fact that these women are recruited in our gynecology clinics which include colposcopy services and are therefore not representative of the overall Puerto Rican population.

Modifying the host innate immune system is a well-known feature of numerous malignancies, including HPV-driven cancers [49]. HPV interferes with the overall inflammatory process through modulating Toll-like receptor (TLRs) signaling and thus cytokine production in the early stages of the immune response [50,51]. Together, cytokines and TLRs identify HPV and encourage immunological signaling such as cell development and differentiation [49,51]. In cervical HPV infections, an impaired immune response to the virus may lead to cervical disease progression [52]. Overall, we found that any HPV infection elevated all cytokine levels but none of the cytokines were significant (S2 Fig). This suggests that HPV infections alone cannot drive cervical dysplasia progression and assure viral persistent infection [53]. Most HR-HPV-infected women do not develop cervical dysplasia because an effective immune response controls the infection and therefore, malignant transformation is halted [54]. Factors promoting a persistent HPV infection can include viral immune evading strategies, such as reducing the amount of IFNγ, altering the activation of antiviral and antitumor immune cells such as NK cells and T cells with antiviral and antitumor activity [55]. Furthermore, a disruption in the mucosal microbiome, characterized by the reduction of beneficial *Lactobacillus* bacteria and associated physicochemical modifications that typically coincide with microbial imbalances, leads to changes in the vaginal and cervical milieu. This situation creates a specific microbial environment that exerts selective pressures on the microbiota [56].

Several studies have already established background data on how the microbiome is modified by HPV infections, stating that HPV infections alone are not enough to drive significant changes that translate into cervical disease progression [16,57]. Nonetheless, other studies suggested changes in the composition of the vaginal microbiota associated with human papillomavirus infection [58–60]. Our results show greater microbiota diversity and a lower abundance of lactobacilli among HPV-positive women (all had cervical disease; LGSIL or HGSIL), which is congruent with previous studies [16,61–63]. It is still unclear why some HR-HPV infections clear while others persist, causing dysplasia and even cervical cancer which can only be evaluated in longitudinal cohorts.

Concerning cervical lesions, we found a strong microbiome association with the cervical phenotype (lesion severity), with a significantly higher diversity value (Shannon) in HGSIL compared to LGSIL participants, but not substantially different from NILM/HPV-. In contrast, richness (Chao1) was also significantly higher in HGSIL when compared to LGSIL and NILM/HPV-. Overall, *Lactobacillus* species have several mechanisms that function as a defensive barrier for invasive bacteria, such as, the production of significant amounts of bacteriocins that contributes to colonization the use of available glycogen to produce lactic acid, lowering vaginal pH, responsible of lysing bacteria other than *Lactobacillus* [64–67]. The loss of these defensive strategies due to the *Lactobacillus* population reduction, may explain the increase of *Atopobium vaginae*, *Prevotella* spp., and *Gardnerella* spp.. Also, our findings coincide with most studies suggesting an association between *L*. *iners* and HGSIL [68,69]. *L*. *iners* exhibits a reduced capacity to consistently maintain the acidic vaginal environment. Conversely, the prevalence of *L*. *crispatus* is linked to higher acid levels, a lower pH, and reduced microbial diversity within the vaginal microbiome. Moreover, research indicates that *L*. *crispatus* displays superior adherence to vaginal epithelial cells, enhancing its ability to outcompete potential pathogens for space and resources within the vaginal environment. In alignment with our findings, previous studies have associated *L*. *crispatus* with the modulation of the local immune response, promoting a well-balanced and healthy microbiome. Conversely, our data demonstrates a correlation between *L*. *iners* and a pro-inflammatory state.

Previous studies have described that *Lactobacillus* can prevent the colonization of exogenous pathogens by producing lactic acid, bacteriocins and reactive oxygen species (ROS) [18,70,71], promoting homeostasis in the cervicovaginal environment. This is the case of *L*. *crispatus* that revealed a significant correlation between high bacterial concentrations and low abundances of pro inflammatory cytokines IL-1β and IFNγ. Our study confirms cervicovaginal microbial dysbiosis in high-risk HPV and cervical disease severity associated with pro-inflammatory cytokines, validating other studies in other cohorts [23,72]. IL-1β was significantly more abundant in high-grade disease participants, associated with a loss in *Lactobacillus* validating previous studies where high concentrations IL-1β were detected in women with cervical neoplasia [73–75]. The rise in IL-1β can be attributed to cellular mechanisms such as tumorigenesis, angiogenesis [76] or metastasis [77]; as well as increased risk of CC in women with high grade cervical lesions [78] associated with either polymorphism [79], reactive oxygen species (ROS), COX2 or NADPH oxidase [80]. We found lower levels of expression of IL-6 associated with low abundance of *L*. *iners*. IL-6 is essential for the growth, metastasis, and initiation of cancer; affecting tumor growth, differentiation, and the host immune defense mechanism [81]. Many immune and nonimmune cells, such as T cells, monocytes, macrophages, dendritic cells, and some cancer cells -such as CC cells- produce IL-6 [82,83]. IL-6 induces CC growth by vascular endothelial growth factor (VEGF)-dependent angiogenesis [84]. In addition, higher expression of IL-6 was shown in cancerous tissues than in adjacent non-cancer tissues in early-stage CC patients, confirming its association to cancer development [85]. The less protective Lactobacilli (*L*. *iners)* may be stimulating the production of these mediators, this could be due in part to their heterogeneous genome, that, unlike the protective counterparts, do not produce as much lactic acid or hydrogen peroxide [86].

Anti-inflammatory cytokine IL-4 was highly elevated on participants with HGSIL. IL-4 within the cervicovaginal microbiome regulates the immune response [87], upholds tissue well-being [88], and indirectly impacts the microbial community. Its significance lies in its anti-inflammatory properties, pivotal in averting undue inflammation prompted by the resident microbiota. Furthermore, it can also facilitate tissue mending and the process of wound healing [88]. Traffic associated cytokine, IL-8, was also highly elevated on participants with HGSIL. IL-8 plays a crucial role in attracting and guiding immune cells, particularly neutrophils, to the site of infection or inflammation [89]. This chemokine is vital for initiating the immune response to combat potential pathogens or respond to tissue damage [90]. In the context of the cervicovaginal microbiome, elevated levels of IL-8 can indicate an ongoing immune response or inflammation due to microbial imbalance or infection in the vaginal environment. Therefore, monitoring IL-8 levels can provide insights into the health and immune status of the cervicovaginal microbiome.

Taxonomic profiles of cervical microbial communities were sorted into community state types (CSTs), allowing a valuable categorization for statistical modeling and comparisons between datasets (18,19). The vaginal microbiota in this Hispanic women cohort of women living in Puerto Rico was dominated by CST IV-C (57.1%, non-*Lactobacillus* profile) and CST IV-A (24.1%, *G*. *vaginalis* moderate), which confirms previous work of our lab [16], and suggests similarities to other US Hispanics [18] and Venezuelans [91]. Even when CST IV-C dominated all three categories of cervical cytology (NILM, LGSIL, HGSIL), but was more predominant in healthy patients (NILM) (62.5%). CST IV-C is characterized by a low relative abundance of *Lactobacillus spp*., *G*. *vaginalis*, and *A*. *vaginae*, while instead is characterized by the abundance of a diverse array of facultative and strictly anaerobic bacteria, thus revealing that Hispanic women living in PR are in constant dysbiosis with a microbiome dominated by anaerobic non-lactobacillus communities. Overall, the high prevalence of CST-IV across all samples reflects a cervical microbial environment with low stability and less likelihood to return to its equilibrium state after a perturbation, which increases the risk of developing adverse health outcomes [92,93]. In Hispanics living in Puerto Rico, the typical healthy vaginal microbial profile seems to be already diverse. It does not correspond to that of US Caucasians, mostly dominated by CST-I, being more like that of other US Hispanics, which are more prone to vaginal dysbiosis regulation and inflammatory conditions [18]. CST-IV represents a highly diverse bacterial profile and has been associated with high pro-inflammatory cytokine levels, which damage the endocervix’s columnar epithelial barrier, exposing it to colonization to other microbial or pathogenic agents [17,94].

Microbiome analysis revealed that high levels of all three groups of cytokines (anti-inflammatory, pro-inflammatory, and traffic-associated) were related to significant bacterial community composition changes and high diversity levels (Shannon). High and low levels of *Sneathia*, associate with higher concentrations of IL-1β. *Sneathia spp*. are regarded as biomarkers for bacterial vaginosis [95], a classical dysbiosis associated with abnormalities in cervical smears [96]. This relationship with pro-inflammatory IL-1β supports the reports that consider these bacteria as pathogens of the female reproductive tract [97]. Increased IL-1β significantly correlated with higher abundances of dysbiosis-associated bacteria, including *Gardnerella*, *Prevotella*, *Atopobium*, and *Sneathia*. This confirms the keystone-pathogen hypothesis with low-abundance taxa associated with dysbiosis and inflammation [98]. A study suggested that *Prevotella* may provide nutrients such as ammonia and amino acids to other microbial community members such as *Gardnerella* and *Peptostreptococcus* [99], assuming a metabolic hub for vaginal microbiota and being a key player in vaginal dysbiosis [100]. *Prevotella* is therefore critical for maintaining a dysbiotic ecosystem in the cervix. Low concentrations of traffic-associated-related cytokines were associated with an increase in *Prevotella* and *Atopobium*, suggesting that there is little induction of immune cell tissue migration to combat these bacteria. Previous studies reported that *Atopobium vaginae* could trigger an innate immune response involving IL-6 and IL-8 using an in vitro model of bacterial vaginosis [101], and this could be happening *in vivo*.

Cervical dysplasia is a complex multifactorial process, and further research, including time-series analyses of longitudinal sampling, is needed to improve our understanding of its etiology. Our study evidence that high concentrations of all three cytokine groups (inflammatory, anti-inflammatory, and traffic-associated) associates with cervical dysbiosis, which occurs through dramatic losses of protective *Lactobacilli* and the presence of diverse anaerobic taxa. We acknowledge that the main limitation of our study is the relatively low sample number, especially in the categorical groups (n = 91), besides lack of information regarding other viral infections and sexual behavior and menstrual health. The cervical microbiota in Hispanic women living in Puerto Rico is highly diverse and dominated by CSTs III and IV. Further changes of the microbiota with HR-HPV and inflammation reveal that the joint host-microbe interaction analyses via cytokine signaling and microbiota in precancerous lesions have great translational potential.

## Supporting information

S1 Fig CST prevalence distribution among cytokine levels and groups. CSTs according to category groups were used to compute Fisher’s test using pairwise analysis to compare between groups. Results were depicted in boxplots for cervical disease. Significant differences are highlighted by brackets and corresponding p-values.
(PDF)

S2 Fig Cytokine Profiles of (A) pro inflammatory (IL-1β, TNFα, IFNγ, IL-6), (B) anti-inflammatory (IL-4, IL-10, TGFβ1) and (C) traffic (IL-8, MIP1a, MCP1, IP10) cytokines evaluated through LUMINEX, plotted according to HPV risk. Cytokine concentrations (pg/mg protein) were used to compute multiple comparison analysis using ordinary one-way ANOVA with Tukey’s multiple comparisons test. Significant differences are highlighted by brackets and corresponding p-values.
(PDF)

S3 Fig Diversity and cytokine profile analyses comparing samples, according to menopause status. Cytokine concentrations (pg/mg protein) were used to compute multiple comparison analysis using ordinary one-way ANOVA with Tukey’s multiple comparisons test (A-C). Beta and alpha diversity analyses are represented by non-metric multidimensional scaling (NMDS) (D) and Shannon index boxplots (E). Relative abundance of bacteria at the species level is shown in a relative abundance bar plot (F).
(PDF)

S4 Fig Diversity analyses comparing samples, according to inflammatory (IL-1β, TNFa, IFNg, IL-6), anti-inflammatory (IL-4, IL-10, TGFβ1), and traffic (IL-8, MIP1a, MCP1, IP10) cytokines. Bray-Curtis analysis represented by Non-metric multidimensional scaling (NMDS), alpha diversity (Shannon) and bar plots showing relative abundance of bacteria at the species level were grouped using pro inflammatory (A), anti-inflammatory (B) and trafficking cytokines (C) as metadata categories.
(PDF)

S1 Table Study population characteristics (n = 91) described by cervical disease status, including HPV risk, community state types, and cytokine ranks. (DOCX)

S2 Table Pairwise comparisons using Pairwise comparison of proportions (Fisher) of the cytokine levels. P values were adjusted using Bonferroni corrections.
(DOCX)

S1 File Inclusivity in global research. (DOCX)
