# Supplementary material for: Inflammatory cytokines and a diverse cervicovaginal microbiota associate with cervical dysplasia in a cohort of Hispanics living in Puerto Rico
Source: PLoS One. 2023 Dec 8;18(12):e0284673. doi: 10.1371/journal.pone.0284673 (PMC10707696; doi:10.1371/journal.pone.0284673)
Supplement: S1 Fig — CSTs according to category groups were used to compute Fisher’s test using pairwise analysis to compare between groups. Results were depicted in boxplots for cervical disease. Significant differences are highlighted by brackets and corresponding p-values. (PDF) [file pone.0284673.s001.pdf]

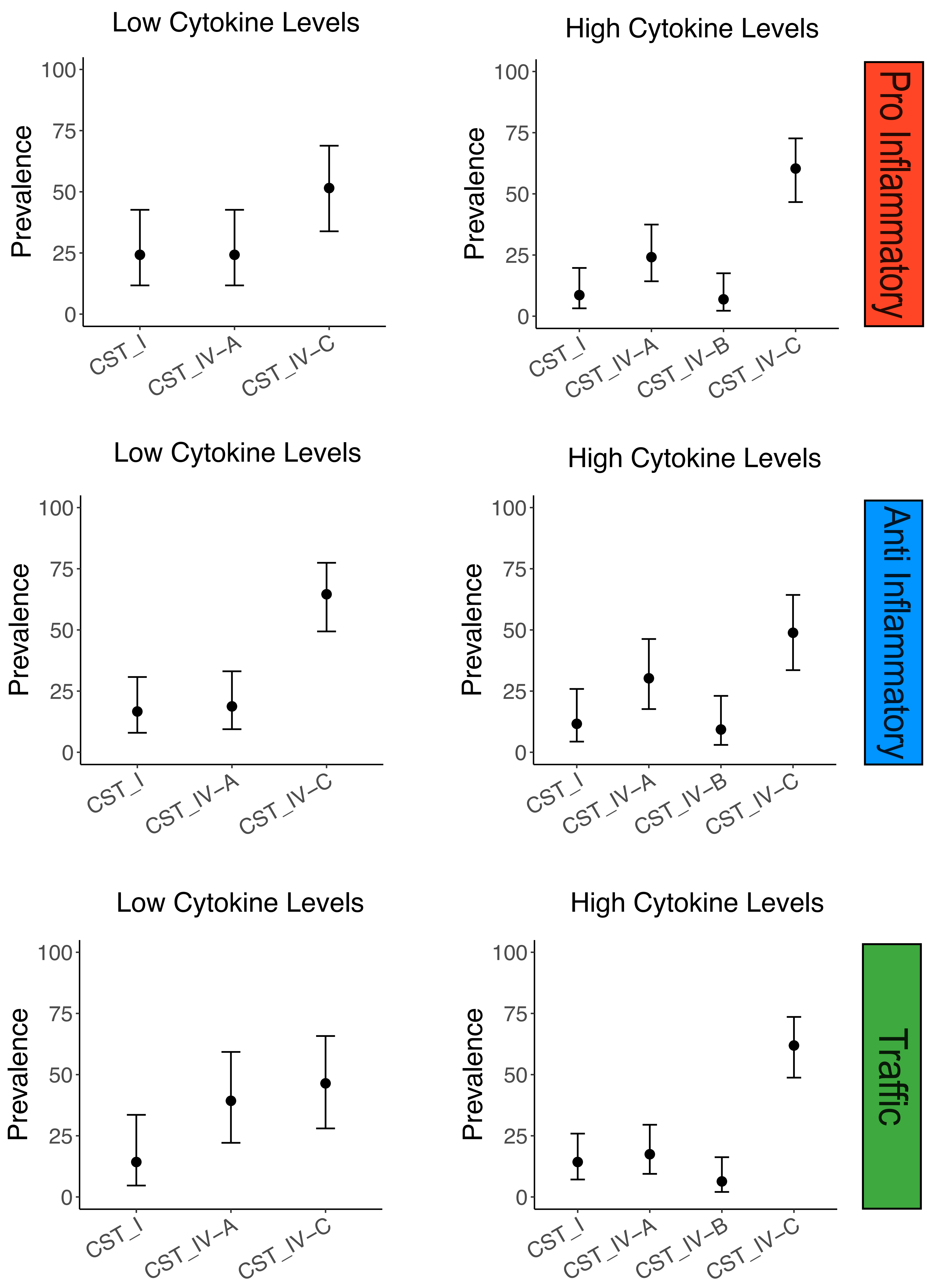

Low Cytokine Levels
High Cytokine Levels
Pro Inflammatory
Anti Inflammatory
Traffic
Prevalence
100
75
50
25
0
CST_I
CST_IV–A
CST_IV–B
CST_IV–C
