# Supplementary material for: Inflammatory cytokines and a diverse cervicovaginal microbiota associate with cervical dysplasia in a cohort of Hispanics living in Puerto Rico
Source: PLoS One. 2023 Dec 8;18(12):e0284673. doi: 10.1371/journal.pone.0284673 (PMC10707696; doi:10.1371/journal.pone.0284673)
Supplement: S2 Fig — Cytokine concentrations (pg/mg protein) were used to compute multiple comparison analysis using ordinary one-way ANOVA with Tukey’s multiple comparisons test. Significant differences are highlighted by brackets and corresponding p-values. (PDF) [file pone.0284673.s002.pdf]

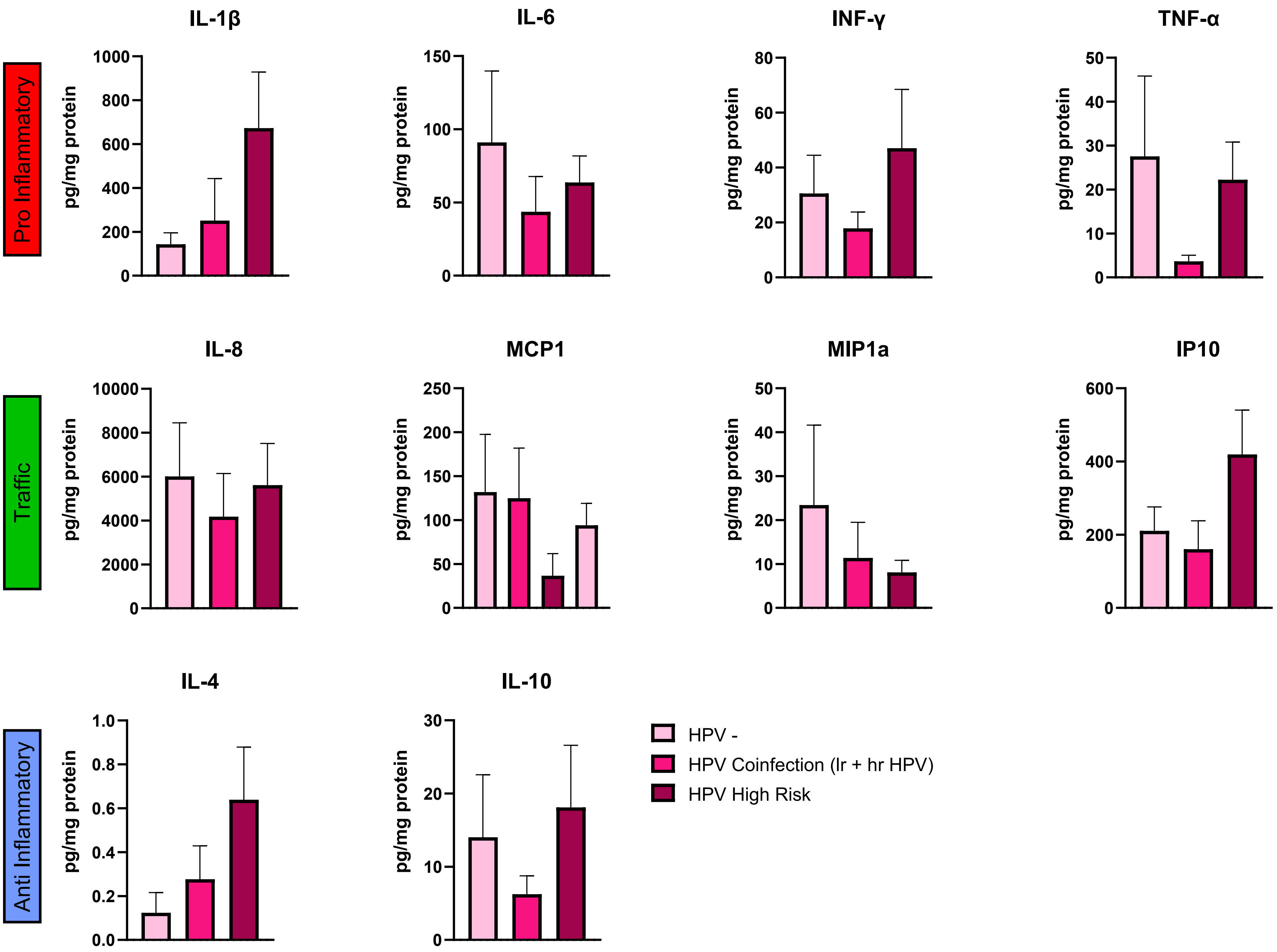

Pro Inflammatory
Traffic
Anti Inflammatory
IL-1β
IL-6
INF-γ
TNF-α
IL-8
MCP1
MIP1a
IP10
IL-4
IL-10
pg/mg protein
HPV -
HPV Coinfection (lr + hr HPV)
HPV High Risk
