# Supplementary figures and images for: Inflammatory cytokines and a diverse cervicovaginal microbiota associate with cervical dysplasia in a cohort of Hispanics living in Puerto Rico
Source: PLoS One. 2023 Dec 8;18(12):e0284673. doi: 10.1371/journal.pone.0284673 (PMC10707696; doi:10.1371/journal.pone.0284673)

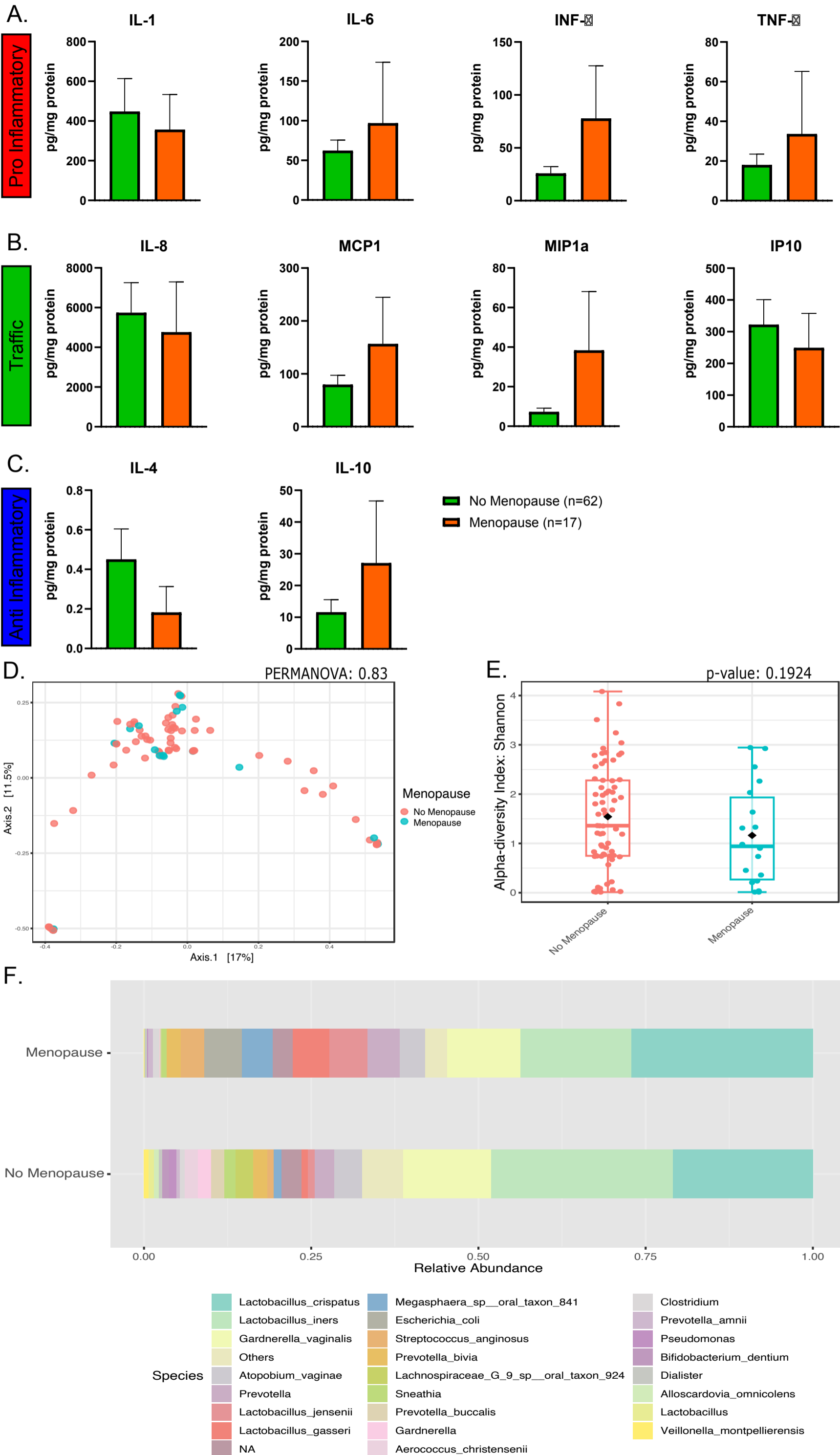

Supplement: S3 Fig — Cytokine concentrations (pg/mg protein) were used to compute multiple comparison analysis using ordinary one-way ANOVA with Tukey’s multiple comparisons test (A-C). Beta and alpha diversity analyses are represented by non-metric multidimensional scaling (NMDS) (D) and Shannon index boxplots (E). Relative abundance of bacteria at the species level is shown in a relative abundance bar plot (F). (PDF) [file pone.0284673.s003.pdf]

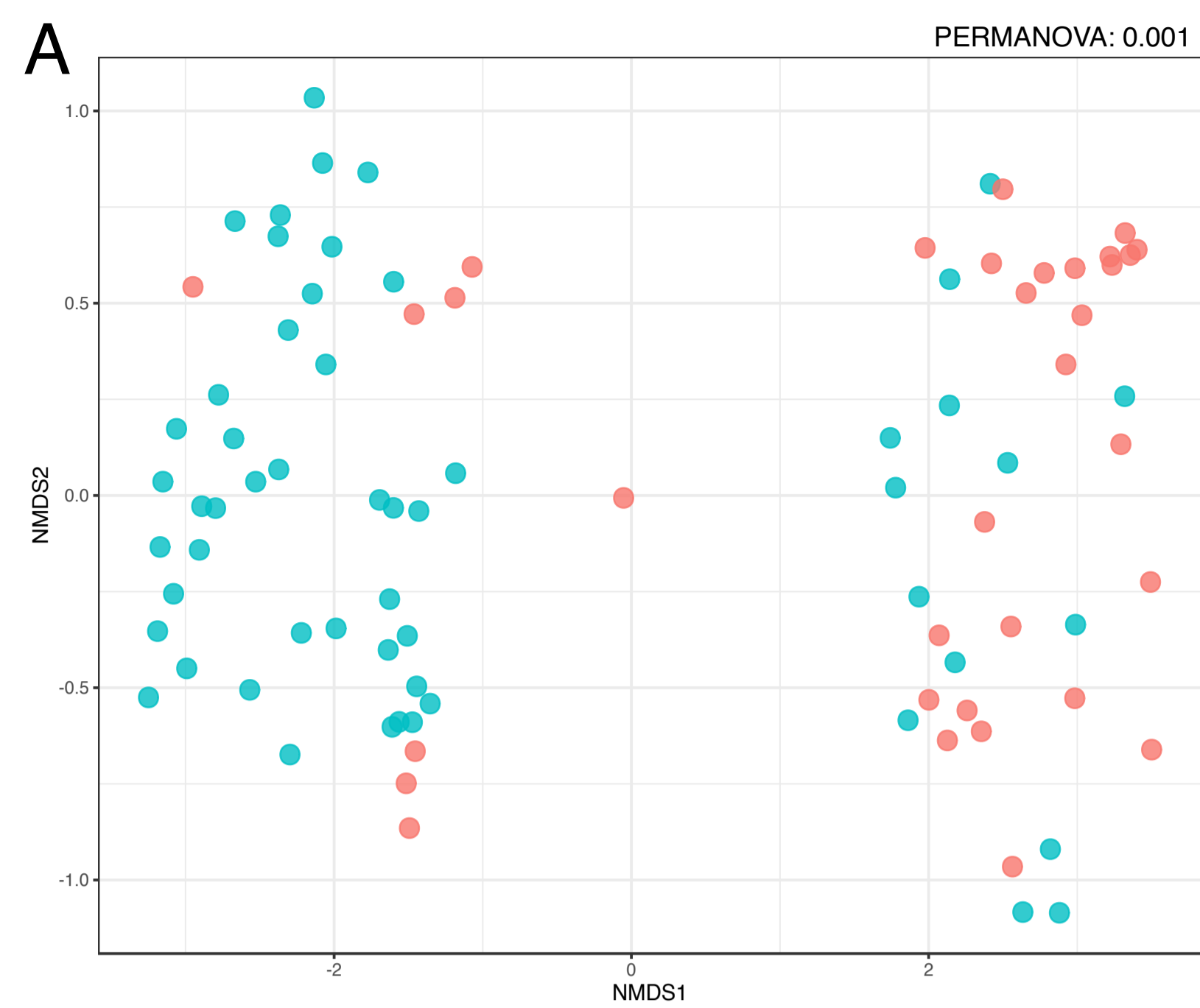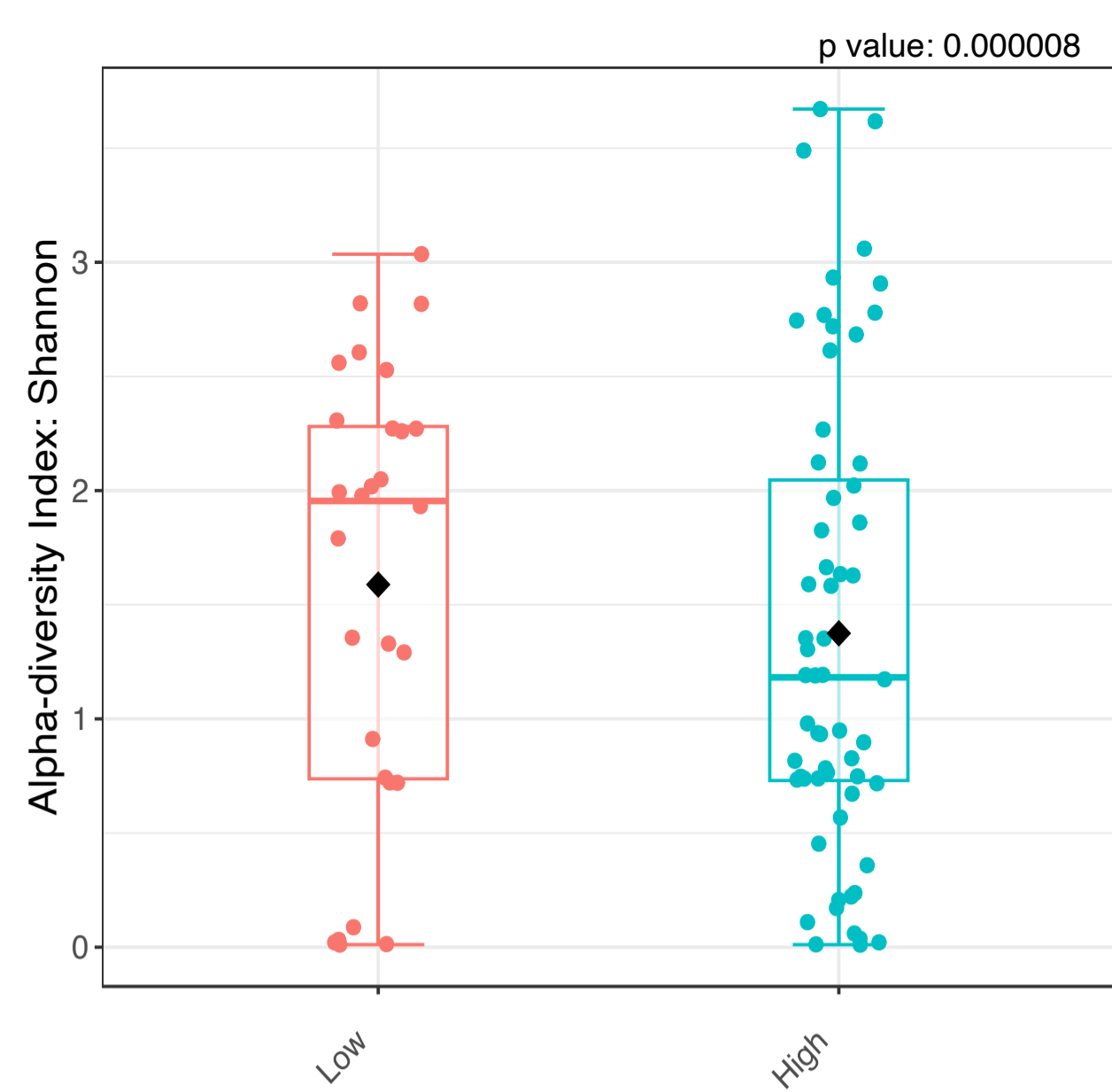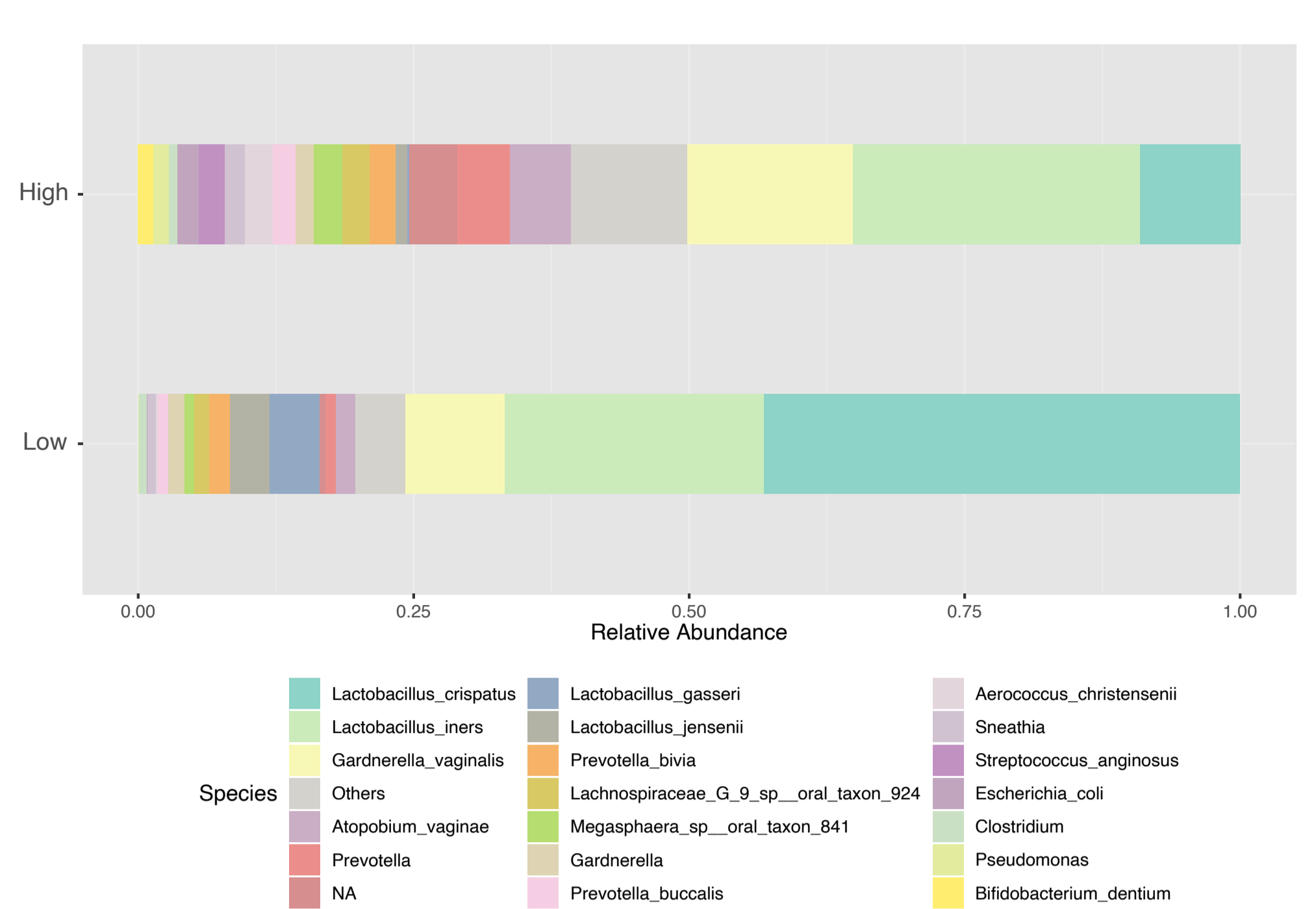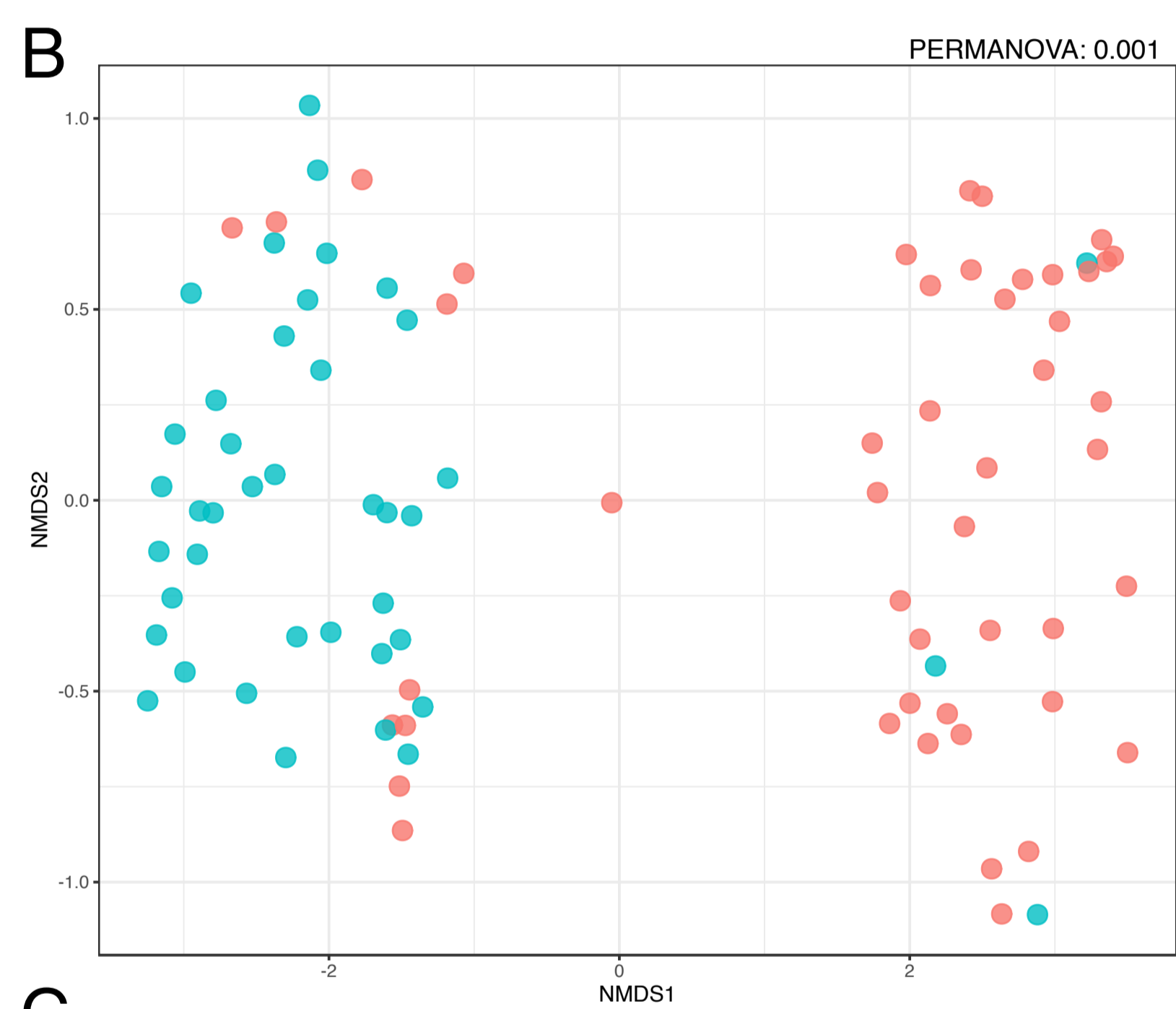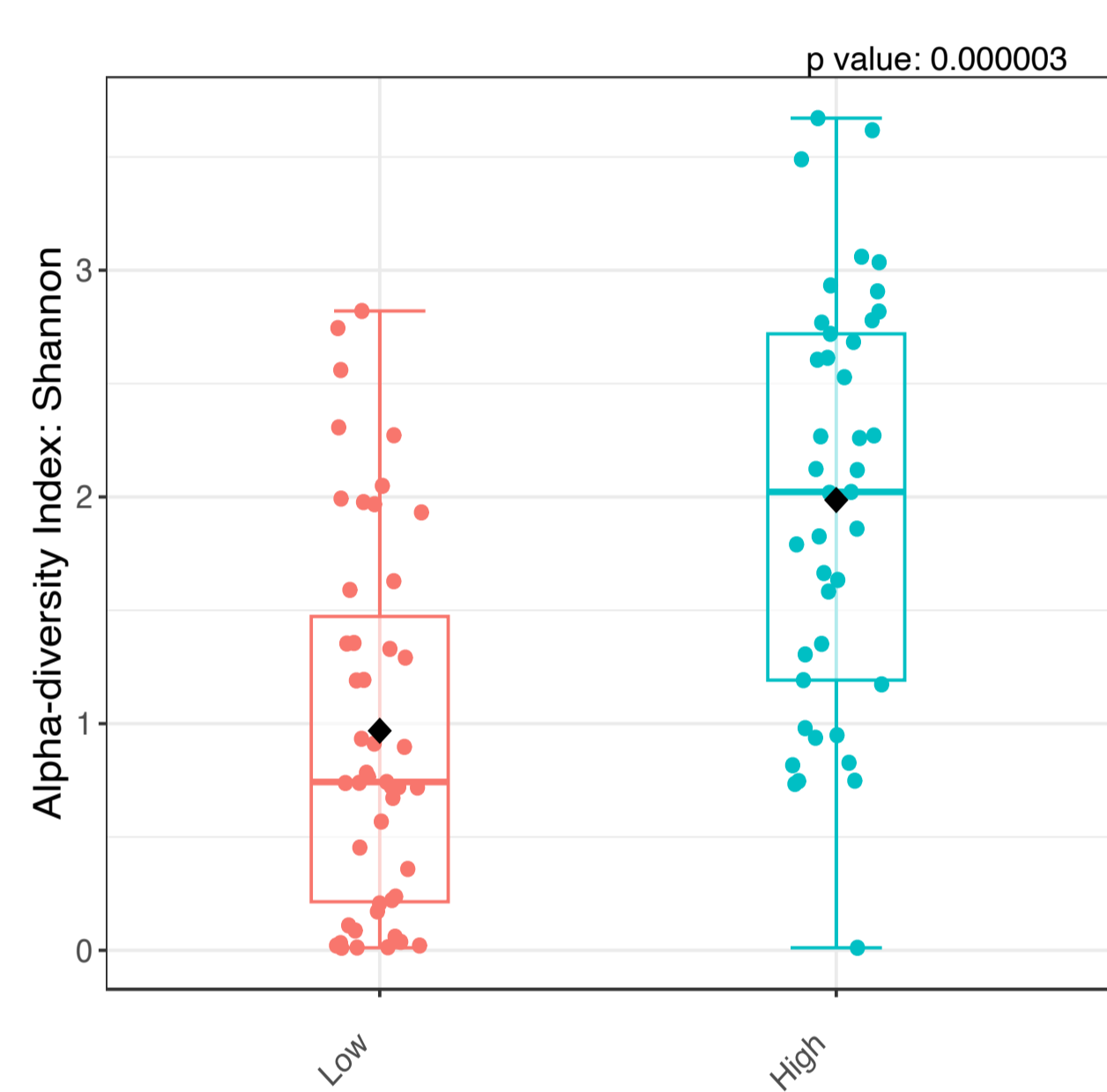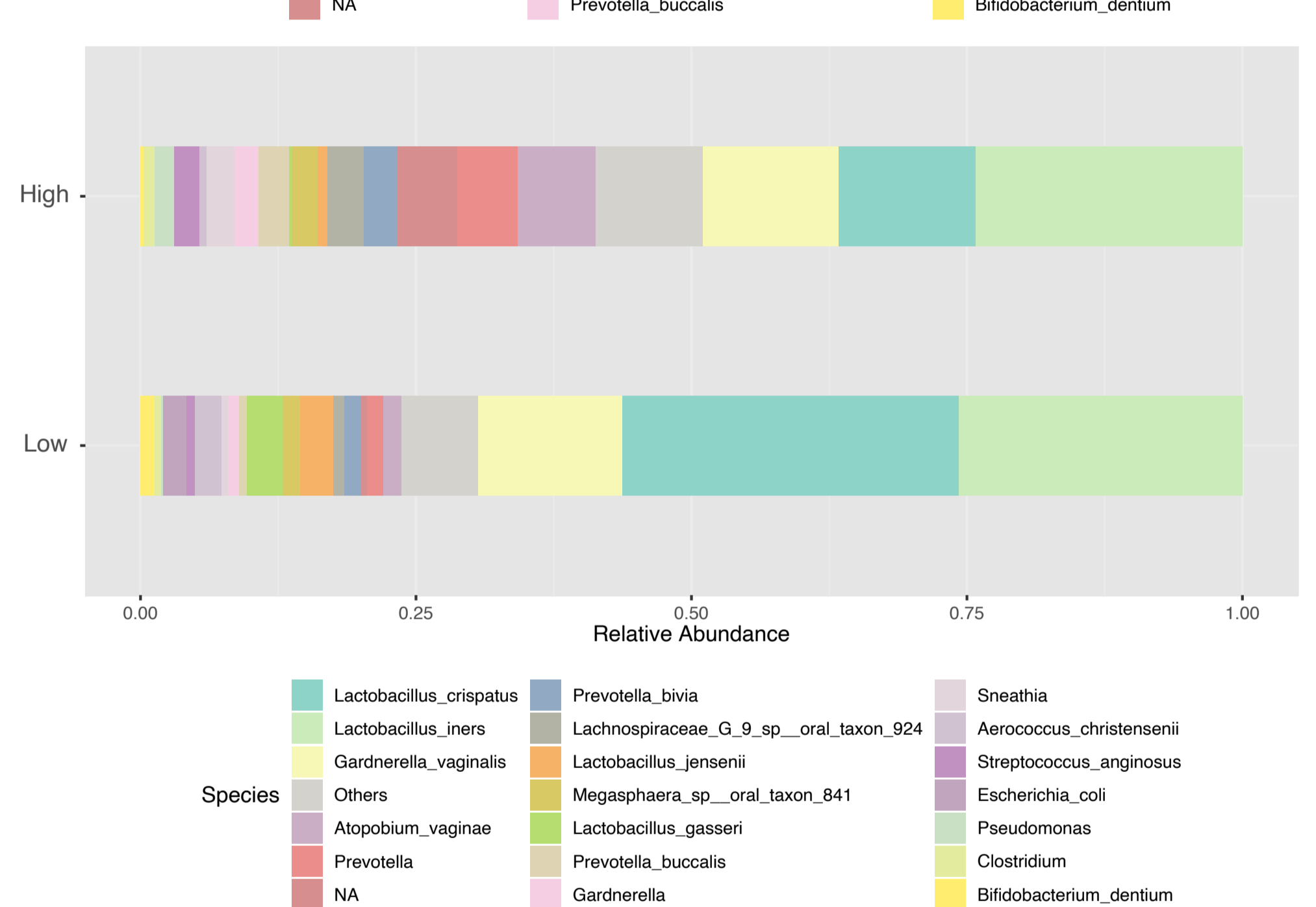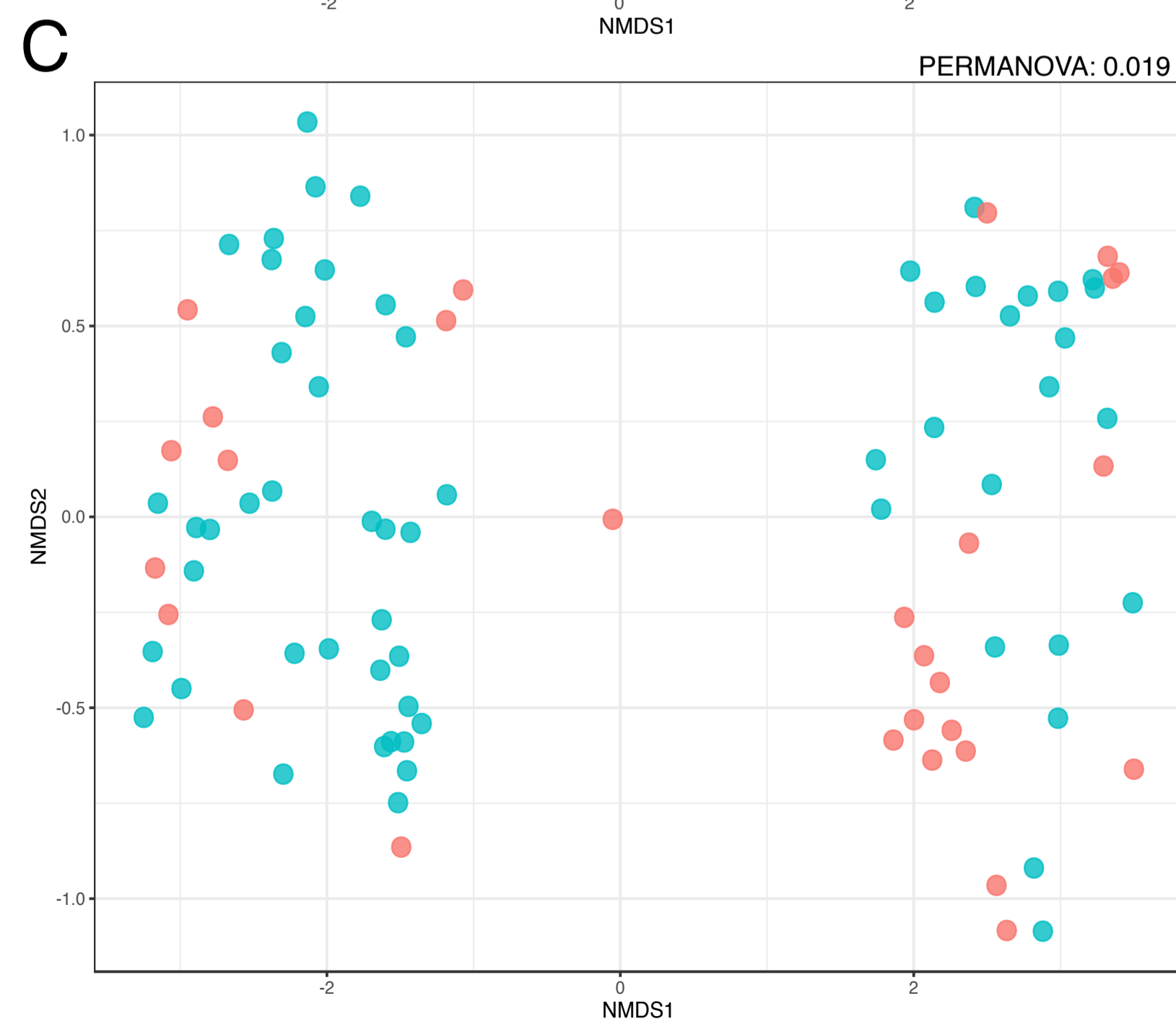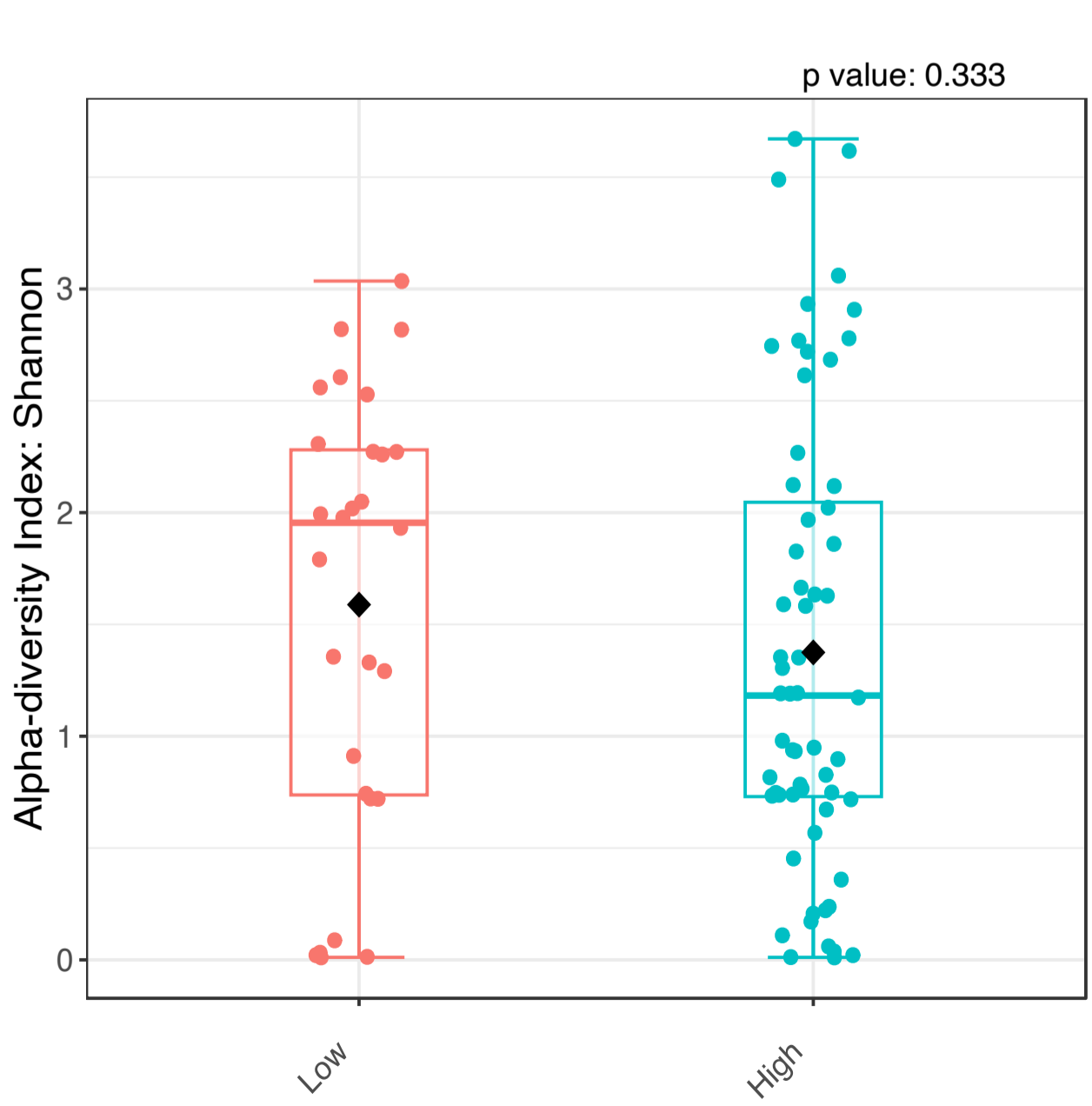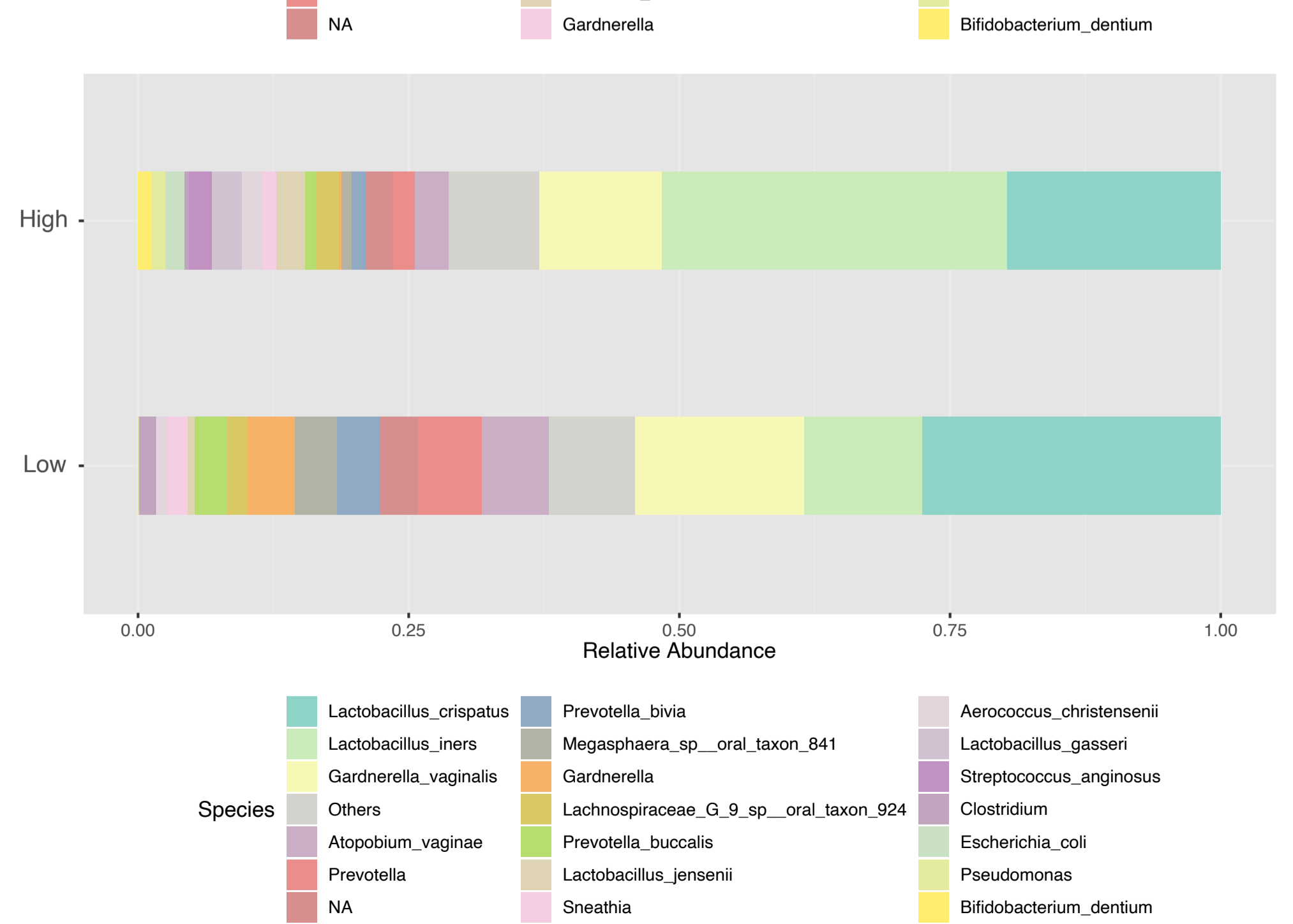

Supplement: S4 Fig — Bray-Curtis analysis represented by Non-metric multidimensional scaling (NMDS), alpha diversity (Shannon) and bar plots showing relative abundance of bacteria at the species level were grouped using pro inflammatory (A), anti-inflammatory (B) and trafficking cytokines (C) as metadata categories. (PDF) [file pone.0284673.s004.pdf]
