# Supplementary material for: Inflammatory cytokines and a diverse cervicovaginal microbiota associate with cervical dysplasia in a cohort of Hispanics living in Puerto Rico
Source: PLoS One. 2023 Dec 8;18(12):e0284673. doi: 10.1371/journal.pone.0284673 (PMC10707696; doi:10.1371/journal.pone.0284673)
Supplement: S1 Table — (DOCX) [file pone.0284673.s005.docx]

**S1 Table. Study population characteristics (n=91) described by cervical disease status, including HPV risk, community state types, and cytokine ranks.**

| **Variable** | **Group** | **N** | **%** | **Average of sequences ± SD** | **Average of ASVs ± SD** |
| --- | --- | --- | --- | --- | --- |
| **Cervical Disease** | NILM/HPV- | 27 | 29.67 | 25839.18 ± 8890.22 | 86.14 ± 165.37 |
|  | NILM/HPV+ | 29 | 31.86 | 24214.62 ± 11665.28 | 100.51 ± 121.35 |
|  | LGSIL | 18 | 19.78 | 19561.67 ± 10061.98 | 241.22 ± 514.80 |
|  | HGSIL | 17 | 18.68 | 18534.76 ± 6478.49 | 169.05 ± 195.67 |
| **HPV Risk** | HPV- | 30 | 32.96 | 25452.10 ± 8597.48 | 84.77 ± 156.66 |
|  | HPV+ Coinfection | 16 | 17.58 | 19723.00 ± 10997.47 | 292.68 ± 536.51 |
|  | HPV+ High Risk | 45 | 49.45 | 21954.48 ± 10347.22 | 116.24 ± 154.06 |
| **Community State Types (CSTs)** | CST I | 13 | 14.29 | 23870.15 ± 9871.72 | 47.23 ± 38.05 |
|  | CST IV-A | 22 | 24.18 | 23472.31 ± 11521.58 | 91.91 ± 70.97 |
|  | CST IV-B | 4 | 4.40 | 20915.25 ± 9463.05 | 130.50 ± 24.14 |
|  | CST IV-C | 52 | 57.14 | 22244.59 ± 9683.67 | 178.82 ± 348.59 |
| **IL-1β Ranks** | Low | 45 | 49.45 | 26039.02 ± 10575.19 | 130.60 ± 337.31 |
|  | High | 46 | 50.54 | 19463.63 ± 8377.74 | 143.04 ± 185.77 |
| **IL-6 Ranks** | Low | 49 | 53.84 | 25607.18 ± 10346.02 | 129.22 ± 323.97 |
|  | High | 42 | 46.15 | 19341.21 ± 8606.23 | 145.83 ± 192.44 |
| **INF-γ Ranks** | Low | 51 | 56.04 | 24826.66 ± 9740.89 | 134.49 ± 317.97 |
|  | High | 40 | 43.95 | 20023.07 ± 9872.38 | 139.95 ± 196.55 |
| **TNF-ɑ Ranks** | Low | 41 | 45.05 | 28725.65 ± 8783.57 | 50.19 ± 46.44 |
|  | High | 50 | 54.94 | 17786.62 ± 8182.70 | 207.98 ± 347.46 |
| **IL-8 Ranks** | Low | 52 | 57.14 | 24094.23 ± 10691.04 | 131.65 ± 313.79 |
|  | High | 39 | 42.85 | 20876.48 ± 8889.16 | 143.87 ± 201.16 |
| **MCP1 Ranks** | Low | 45 | 49.45 | 26554.42 ± 10325.71 | 140.88 ± 337.92 |
|  | High | 46 | 50.54 | 18959.43 ± 8245.09 | 132.97 ± 184.81 |
| **MIP1a Ranks** | Low | 46 | 50.54 | 21561.30 ± 10502.64 | 164.84 ± 349.71 |
|  | High | 45 | 49.45 | 23894.73 ± 9504.80 | 108.31 ± 149.74 |
| **IP10 Ranks** | Low | 38 | 41.75 | 29860.34 ± 7992.71 | 48.55 ± 42.25 |
|  | High | 53 | 58.24 | 17592.26 ± 8047.88 | 200.22 ± 339.33 |
| **IL-4 Ranks** | Low | 48 | 52.74 | 26145.95 ± 10149.61 | 71.25 ± 69.89 |
|  | High | 43 | 47.25 | 18885.51 ± 8487.09 | 210.16 ± 374.66 |
| **IL-10 Ranks** | Low | 42 | 46.15 | 27968.30 ± 9817.80 | 132.00 ± 349.72 |
|  | High | 49 | 53.84 | 18212.53 ± 7850.47 | 141.08 ± 179.48 |
| **TGF-β1 Ranks** | Low | 47 | 51.64 | 28216.57 ± 9699.22 | 123.06 ± 331.33 |
|  | High | 44 | 48.35 | 16838.72 ± 6457.84 | 151.65 ±186.40 |
