# Supplementary material for: Inflammatory cytokines and a diverse cervicovaginal microbiota associate with cervical dysplasia in a cohort of Hispanics living in Puerto Rico
Source: PLoS One. 2023 Dec 8;18(12):e0284673. doi: 10.1371/journal.pone.0284673 (PMC10707696; doi:10.1371/journal.pone.0284673)
Supplement: S2 Table — P values were adjusted using Bonferroni corrections. (DOCX) [file pone.0284673.s006.docx]

**S2 Table.** Pairwise comparisons using Pairwise comparison of proportions (Fisher) of the cytokine levels. P values were adjusted using Bonferroni corrections.

| **Cytokines** | **Low Cytokine Levels** | | | **High Cytokine Levels** | | | |
| --- | --- | --- | --- | --- | --- | --- | --- |
| **Pro Inflammatory** |  | CST_I | CST_IV_A |  | CST_I | CST_IV_A | CST_IV_B |
|  | CST_IV_A | 1 | - | CST_IV_A | 0.256 | - | - |
|  | CST_IV_C | 0.12 | 0.12 | CST_IV_B | 1 | 0.114 | - |
|  | - | - | - | CST_IV_C | **2.30E-8** | **0.001** | **4.1E-09** |
|  |  | CST_I | CST_IV_A |  | CST_I | CST_IV_A | CST_IV_B |
| **Anti-Inflammatory** | CST_IV_A | 1 | - | CST_IV_A | 0.369 | - | - |
|  | CST_IV_C | **8.90E-6** | **2.80E-5** | CST_IV_B | 1 | 0.169 | - |
|  | - | - | - | CST_IV_C | **0.002** | 0.732 | **0.001** |
| **Traffic** |  | CST_I | CST_IV_A |  | CST_I | CST_IV_A | CST_IV_B |
|  | CST_IV_A | 0.205 | - | CST_IV_A | 0.369 | - | - |
|  | CST_IV_C | 0.056 | 1 | CST_IV_B | 1 | 0.169 | - |
|  | - | - | - | CST_IV_C | **0.002** | 0.732 | **0.001** |
